# Global Analysis of Mouse Polyomavirus Infection Reveals Dynamic Regulation of Viral and Host Gene Expression and Promiscuous Viral RNA Editing

**DOI:** 10.1371/journal.ppat.1005166

**Published:** 2015-09-25

**Authors:** Seth B. Garren, Yuvabharath Kondaveeti, Michael O. Duff, Gordon G. Carmichael

**Affiliations:** Department of Genetics and Genome Sciences, UCONN Health, Farmington, Connecticut, United States of America; National Institute of Allergy and Infectious Diseases, National Institutes of Health, UNITED STATES

## Abstract

Mouse polyomavirus (MPyV) lytically infects mouse cells, transforms rat cells in culture, and is highly oncogenic in rodents. We have used deep sequencing to follow MPyV infection of mouse NIH3T6 cells at various times after infection and analyzed both the viral and cellular transcriptomes. Alignment of sequencing reads to the viral genome illustrated the transcriptional profile of the early-to-late switch with both early-strand and late-strand RNAs being transcribed at all time points. A number of novel insights into viral gene expression emerged from these studies, including the demonstration of widespread RNA editing of viral transcripts at late times in infection. By late times in infection, 359 host genes were seen to be significantly upregulated and 857 were downregulated. Gene ontology analysis indicated transcripts involved in translation, metabolism, RNA processing, DNA methylation, and protein turnover were upregulated while transcripts involved in extracellular adhesion, cytoskeleton, zinc finger binding, SH3 domain, and GTPase activation were downregulated. The levels of a number of long noncoding RNAs were also altered. The long noncoding RNA MALAT1, which is involved in splicing speckles and used as a marker in many late-stage cancers, was noticeably downregulated, while several other abundant noncoding RNAs were strongly upregulated. We discuss these results in light of what is currently known about the MPyV life cycle and its effects on host cell growth and metabolism.

## Author Summary

Mouse polyomavirus (MPyV) is a small 5.3kb circular double-stranded DNA virus capable of causing tumors in a variety of tissues in immunocompromised mice. It has been a subject of study for over 60 years, yielding insights into a number of processes including tumorigenesis, cell cycle signaling, and transformation. This study serves to provide a global view of the MPyV infection by utilizing Illumina sequencing to observe changes in total RNA from both the virus and the host cell as well as applying new methods to more directly confirm the extent of A-to-I editing of viral RNA by host ADAR enzymes. This allows for a simultaneous observation of both host and viral transcriptional changes that occur as a result of early gene expression and the viral switch from early to late genes that occurs coincident with the initiation of DNA replication.

## Introduction

**Fig 1:**
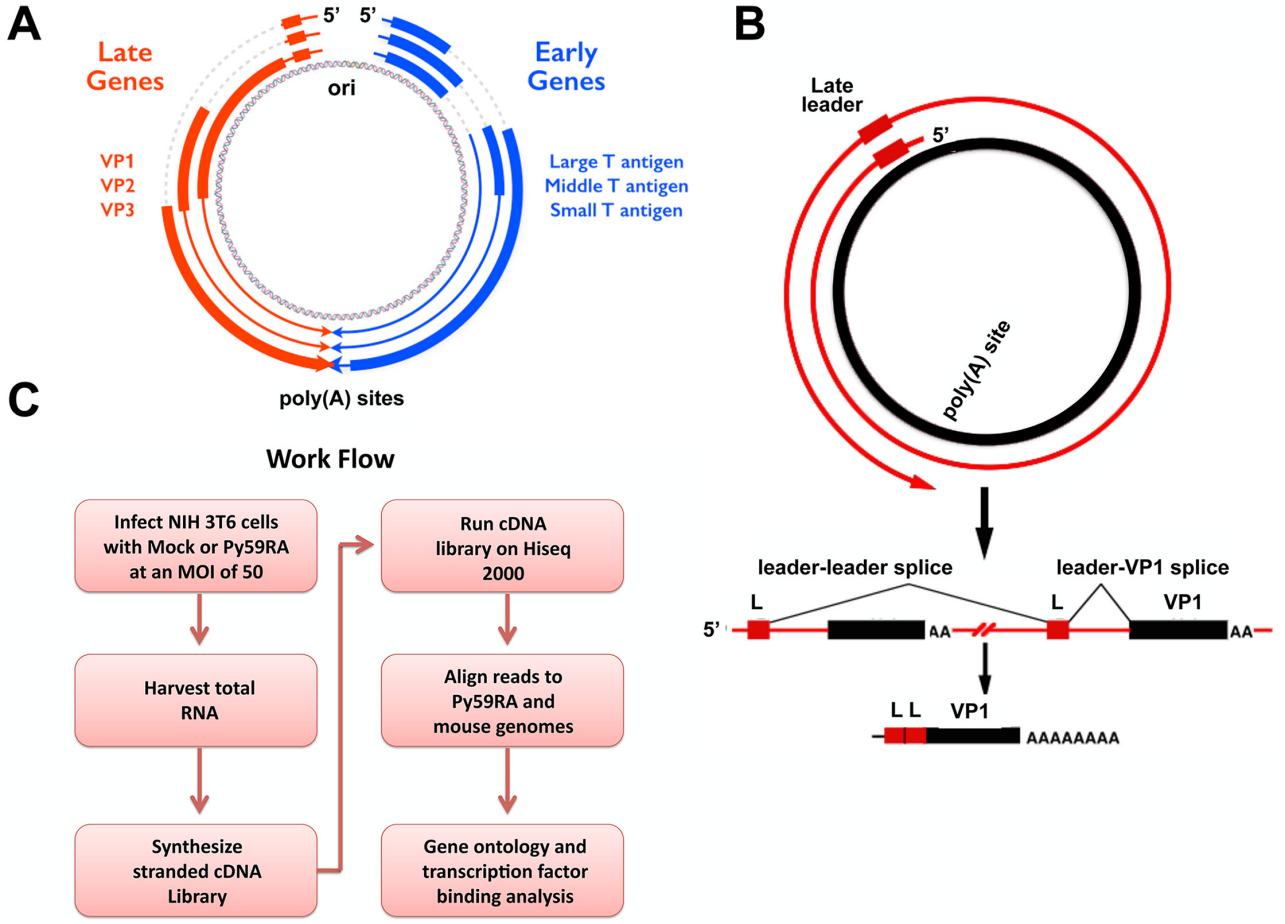
Polyomavirus genome and work flow. **(A)** Polyomavirus genome including origin and polyadenylation sites. Early gene splicing shown in blue. Late gene splicing shown in red. **(B)** Schematic of read-through of the polyadenylation site during the late phase of infection. Late transcripts must read through the entire viral genome at least once to allow for the late leader exon (L) to splice properly. This results in spliced late mRNAs with at least two tandem repeats of the late leader exon. **(C)** Work flow of experiments. NIH 3T6 cells were infected with the Py59RA strain of polyomavirus and either harvested at different time points or treated with aphidicolin to block DNA replication and keep the infection in the early phase for 48 hours. Total RNA was collected and used to synthesize stranded cDNA libraries using the Illumina TruSeq Stranded Total RNA Preparation kit. Samples were run on the Hiseq 2000 sequencer and aligned to both the Py59RA and mouse host genomes.

MPyV is a small circular double-stranded DNA virus with a life cycle that is divided into distinct early and late phases of infection. During the early phase the virus expresses both early-strand and late-strand transcripts from its bidirectional noncoding control region (Fig 1A), but the early-strand transcripts preferentially accumulate and are spliced into mRNAs for Large, middle, and small tumor antigens [1–9]. These early gene products induce host cell S phase entry and the virus utilizes host factors for viral DNA replication [10]. Around 12–16 hours after infection, DNA replication begins, late-strand RNAs accumulate rapidly, and are alternatively spliced to generate mRNAs for the viral capsid proteins VP1, VP2, and VP3 [6,11,12].

MPyV Large T antigen is involved in a variety of roles during the course of infection, including altering host protein pathways to promote virus production [13], blocking the interferon antiviral response [14], recruiting host DNA replication factors to the viral origin [15,16,17], acting as a helicase for viral DNA replication [18], and modulating late gene transcription [19]. Expression of MPyV Large T antigen is sufficient to immortalize primary rat embryonic fibroblasts [20]. Large T affects host cell cycle progression by binding to proteins in the retinoblastoma family of tumor suppressors, pRB, p107, and p130 [21,22] as well as CREB binding protein (CBP) and p300, which regulate cell growth, transformation, and development [23,24]. All three T antigens share an N-terminal DnaJ domain, which can bind the heat shock protein Hsc70 and induce its ATPase function [25]. Large T antigen exhibits both J domain dependent and independent binding to pRB [26].

MPyV middle T antigen is a highly oncogenic protein that is necessary for viral transformation of cells in culture. Middle T has no catalytic domain but rather acts as a scaffold, which functions like a constitutively active receptor tyrosine kinase to activate signal transduction pathways such as Shc, PI3K, and PLCγ [27]. Middle T contains a hydrophobic C-terminal region that anchors it to the cell membrane, which is necessary for it to function [28]. Both middle T and small t contain a domain that binds the A and C subunits of PP2A by mimicking its B subunit [29,30]. Once middle T binds to PP2A it can then bind to protein tyrosine kinases of the Src family such as Src, Yes, and Fyn [31–33]. This causes middle T to be phosphorylated at tyrosine 250, 315, and 322 [34–37], which allows activation of the Shc, PI3K, and PLCγ signaling pathways, respectively [38–40].

MPyV small t antigen functions primarily by binding protein phosphatase 2A (PP2A). Like middle T, small t binds PP2A by mimicking the B subunit to bind to the A and C subunits. MPyV small t is able to bind to both the Aα and Aβ subunits at the B binding site, precluding PP2A regulation by the B subunit [29,41]. Small t has been shown to induce phosphorylation and degradation of the cyclin dependent kinase inhibitor p27 in a PP2A dependent manner, which supports S phase induction [42]. MPyV small t and a mutated form of middle T that does not localize to the cell membrane (and thus cannot interact with Src family kinases) both activate the MAP kinase cascade via PP2A [43]. While Large T antigen does not bind p53 directly in MPyV-infected cells, small and middle T antigens have been shown to resist apoptosis in p53-induced cells through PP2A and PI3K respectively [44].

Although the switch from early to late phase of infection has been thought by some to be regulated primarily at the level of transcription [19], this has been challenged by results consistent, rather, with a change in the processing of late-strand transcripts [1,45]. While the precise mechanism remains to be elucidated, it has been demonstrated that the early-late switch is DNA replication-dependent and results in a reduction of polyadenylation efficiency [3,46]. During the late phase of infection, giant multigenome-length pre-mRNAs from both strands of the viral genome can be detected [47,48]. These giant pre-mRNAs allow for 57 nucleotide late leader exons to splice to one another across genome-length introns. This splicing increases the stability of late transcripts [49] and generates late mRNAs with multiple tandem leader exons at their 5’-ends (Fig 1B) [2,11,50]. The presence of giant transcripts antisense to the early genes also results in sense-antisense RNA duplexes to form, followed by extensive editing by the enzyme Adenosine Deaminase that Acts on RNA (ADAR1), which binds to double stranded RNA and deaminates adenosines to inosines [51,52]. The loss of polyadenylation and transcription termination efficiency that leads to the formation of giant transcripts may be caused, at least in part, by dsRNA or RNA editing induced by the overlap of early-strand and late-strand primary transcripts owing to overlapping polyadenylation signals. Viral mutants that do not allow efficient transcript overlap are defective in their early-late switch [46].

In order to further characterize MPyV infection we have used deep sequencing to compare changes in the transcriptome of both viral and host transcripts at different times of infection (Fig 1C). This allowed strand-specific quantification of the relative changes in transcription, splicing, and editing that occurs during MPyV infection. In addition, a number of new insights into viral gene regulation were obtained, including changes in apparent start sites of early transcripts and extensive RNA editing throughout the viral genome. By 36 hours after infection, 359 genes were shown to be significantly upregulated and 857 genes were shown to be significantly downregulated across three biological replicates when compared to mock infections. Gene ontology analysis showed the upregulated genes to be primarily involved in metabolism, DNA replication, RNA processing, and translation while the downregulated genes were primarily involved in transcription regulation, cell adhesion, extracellular matrix, and signal transduction. Analysis of common transcription factor binding sites showed regulation by transcription factors such as E2F, CREB, ATF, and NFkB in the upregulated genes and SP1, WT1, AP2, and TFIII in downregulated genes.

## Results

### RNA expression in NIH 3T6 cells infected with the Py59RA strain of mouse polyomavirus

Mouse NIH 3T6 cells were chosen for infection for their ability to facilitate lytic infection by wild type MPyV [53]. Cells were serum starved to partially synchronize their cell cycles and then infected without (mock infection) or with filtered Py59RA virus (MPyV) at a multiplicity of infection (MOI) of 50 plaque forming units per cell. The viral titer was determined by plaque assay and was generously provided by Dr. R. Garcea. An MOI of 50 resulted in maximal infection in our system, as measured by immunofluorescence for large T antigen (S1 Fig). Any increase in MOI beyond this point did not result in more infected cells. Total RNA was harvested at 12, 18, 24, and 36-hours post infection from three biological replicates for each condition and was used in the synthesis of stranded cDNA sequencing libraries using the Illumina TruSeq Stranded Total RNA Sample Preparation Kit. These timepoints were chosen because in previous work in our lab we found that the early-late switch occurred between 12 and 16 hours post infection [2,3]. Thus, we selected a timepoint before the onset of DNA replication, one at about the time of its onset and two later in the infection, to allow the accumulation of late viral RNAs and affected cellular transcripts. This resulted in libraries ~260 base pairs in length (S2 Fig). The libraries were sequenced using the Illumina HiSeq 2000 platform using paired-end reads of 100 bases in the forward and reverse directions. Sequencing data was aligned using TopHat and normalized transcript abundance was calculated in fragments per kilobase of exon per million fragments mapped (FPKM) using Cufflinks. Significant differences between the virus and mock infections at each time point were determined using Cuffdiff. Each condition had three biological replicates producing 10–50 million 100 base reads with close to 80% aligning to the host genome and over 1% aligning to the viral genome by 36 hours post infection (S1 Table).

### The mouse polyomavirus transcriptome

**Fig 2:**
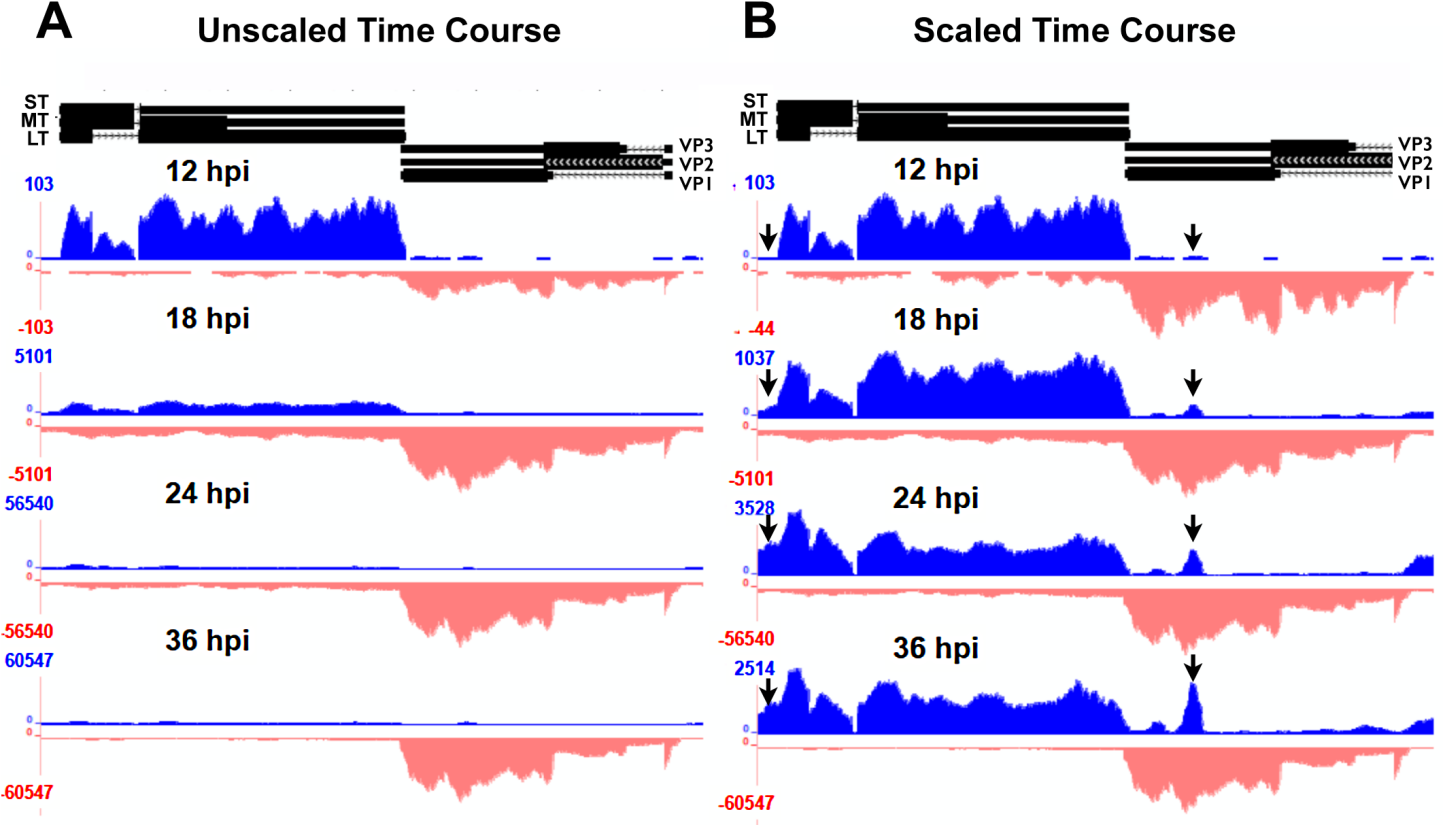
Alignment of time course reads to the Py59RA genome. NIH 3T6 cells were infected with Py59RA at an MOI of 50 plaque forming units/cell or with a mock infection. Total RNA was harvested at 12, 18, 24, and 36 hours and stranded cDNA libraries were prepared for sequencing on the HiSeq 2000 sequencer. Reads were aligned to the Py59RA genome and visualized on the UCSC genome browser. **(A)** Time course reads aligned to the Py59RA genome unscaled to show differential expression between early (plus) and late (minus) strands. **(B)** Time course reads scaled to show changes in early alignment.

In order to visualize changes in viral transcripts at different time points during infection, we used Bowtie and Top Hat to align sequencing reads to a Py59RA reference genome (S3 Fig). At 12 hours after infection, reads mapped to both early and late strands, but with most representing early-strand expression. While this is consistent with a model where the early promoter dominates before the onset of viral DNA replication [19], it could also be consistent with a model in which the early-late switch is dependent on relative transcript stability rather than on regulation of transcription initiation [1]. By 18 hours after infection viral replication has commenced and the infection is entering the late phase. At this point, there is an approximate 100-fold increase in reads aligning to the late strand and these transcripts are beginning to dominate over early-strand transcripts (Fig 2A). Also of note at this time is the appearance of a significant number of transcripts that extend beyond the late polyadenylation site, representing multi-genomic late-strand transcripts [46,47]. While most fully polyadenylated and spliced late RNAs are cytoplasmic [54,55], these giant RNAs are nuclear and serve as precursors to late mRNAs [55–57]. By 24 and 36 hours after infection the number of late strand alignments increases to around 1000 fold compared to early times. We note that at late times the majority of fully processed viral mRNAs have accumulated in the cytoplasm [2,5,7,9].

**Fig 3:**
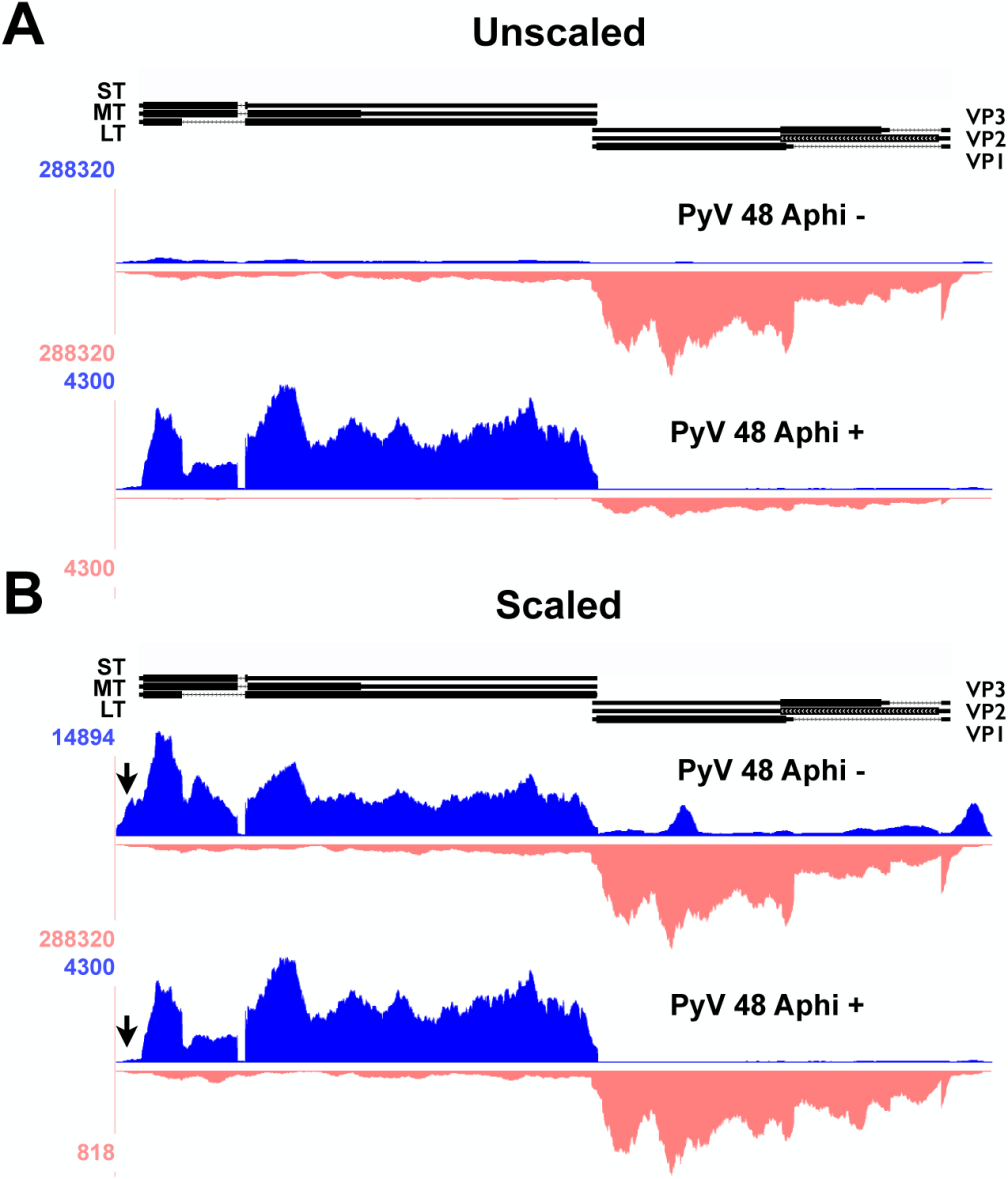
Alignment of 48 hour aphidicolin + and - reads to Py59RA genome. NIH 3T6 cells were infected with Py59RA at an MOI of 50 pfu/cell or with a mock infection and treated with or without the DNA replication inhibitor aphidicolin at a concentration of 2μg/ml (5.9μM). Total RNA was harvested at 48 hours and stranded cDNA libraries were prepared for sequencing on the HiSeq 2000 sequencer. Reads were aligned to the Py59RA genome and visualized on the UCSC genome browser. **(A)** Samples of 48 hr infections with and without the DNA replication inhibitor aphidicolin to block the initiation of late phase aligned to the Py59RA genome unscaled to show differential expression between early and late strands. **(B)** Scaled to show changes in early strand alignment.

While the relative levels of early-strand and late-strands change dramatically during infection, we noted no significant changes in patterns of RNA splicing, but did observe several interesting and unexpected changes to early-strand RNA accumulation during infection. First, it is clear that, relative to the bulk of early-strand RNAs, there is an apparent elevated alignment to a roughly 200 base pair region just downstream of the early polyadenylation site (Fig 2B). This peak was not apparent when DNA replication and the early-late switch was blocked by aphidicolin (Fig 3). In this experiment, mouse NIH3T6 cells were infected with Py59RA virus in the same manner as the time course except they were treated with either the same media or media with 2 μg/ml of the DNA replication inhibitor aphidicolin to prevent the infection from entering the late phase [3]. The infected samples, both treated and untreated, were incubated for 40 and 48 hours before RNA was collected for stranded cDNA library synthesis using the same protocol as the time course. This allowed sufficient accumulation of early gene products without the untreated samples reaching the lysis phase of the viral life cycle. Results were only aligned to the viral genome since aphidicolin treatment would likely cause significant alterations to the host transcriptome.

The second unexpected change in early-strand RNA alignments during infection is in the early promoter/origin/noncoding control region. We originally noted changes in alignments to the edges of our reference genome. By aligning the reads to a linearized reference genome shifted by about 150 bp, we were better able to visualize the nature of these molecules, which span the replication origin. As infection proceeds, there is a clear increase in the alignment of early-strand reads to the origin of replication and enhancer region, strongly suggesting a potential upstream shift in early-strand transcription start sites during infection, and particularly after the onset of viral DNA replication (Fig 2B). We note that even at late times after infection in the aphidicolin treated samples, this shift was not observed (Fig 3B **).** Such a shift from a strict early transcription start site downstream of a canonical TATA box at early times (e.g., the 12 hour time point) to variable upstream transcription start sites at late times was suggested previously to explain confounding results obtained by S1 nuclease experiments [58,59]. Mapping of the hybridization of 5’ ends of cytoplasmic early-strand transcripts at late times to a region of the MPyV genome containing the noncoding control region and part of the early region showed an apparent increase in usage of upstream start sites [58,59]. In order to confirm a shift in early startsites after the onset of DNA replication we performed 5’-RACE analysis. Results (S4 Fig) clearly show that at 12 hrs post infection, most early 5’-ends map to the annotated startsite region, but by 36 hrs post infection, many early-strand transcripts initiate over a broad region between the replication origin and the late promoter startsite region. This replication-dependent switch is not unprecedented, as altered early-strand startsites at late times have been reported both for SV40 [60] and for JC virus [61]. Importantly, Fenton and Basilico reported a broad region of early strand startsites at late times in infection that mapped to sites completely consistent with our data [59].

### Splicing analysis

Using TopHat to map the splice junction reads we were able to determine the relative expression levels of the 5 viral isoforms that contain splice junctions (Table 1). At 12 hours post infection the mRNA for Large T represents 75% of early-strand mRNAs, with mRNAs for middle T and small t accounting for 20% and 5%, respectively. We also note that a fourth early mRNA, for “tiny t antigen” encoding just the DnaJ domain [62] has been reported and this is seen in our data, although this message is of very low relative abundance. As infection proceeds, the ratios of Large, middle and small T antigen mRNAs change little, indicating that early strand splicing is likely not regulated strongly by viral or host proteins that change during infection. Like early mRNAs, late splicing patterns do not change dramatically during infection, although the absolute numbers of alignments increase up to 1000 fold, consistent with the strong upregulation of late gene expression. The ratio of spliced isoforms in the aphidicolin treated infections was essentially the same as in the time course experiment (large T 59%, middle T 24%, small t 16%, tiny t 1%).

**Table 1:** Viral RNA splicing events during infection.

	Large T	middle T	small t	tiny t	VP1	VP3
12 Hours	54.0(±16.4)	10.0(±2.6)	3.3(±1.5)	0.0(±0.0)	26.0(±17.8)	4.7(±1.5)
18 Hours	397.7(±173.7)	105.0(±61.8)	99.3(±64.1)	0.0(±0.0)	1552.0(±1029.1)	278.0(±64.2)
24 Hours	1182.7(±458.8)	367.3(±169.5)	286.7(±83.8)	7.7(±2.1)	24424.3(±14679.3)	4085.3(±510.2)
36 Hours	953.3(±760.4)	333.7(±225.5)	236.0(±182.0)	9.0(±1.7)	22355.0(±15109.8)	3869.7(±969.8)

Average and standard deviation of aligned reads spanning viral splice junctions during the time course across three biological replicates. Percentages shown are with respect to total early or total late splice junction alignments.

### Leader-to-leader splicing

**Fig 4:**
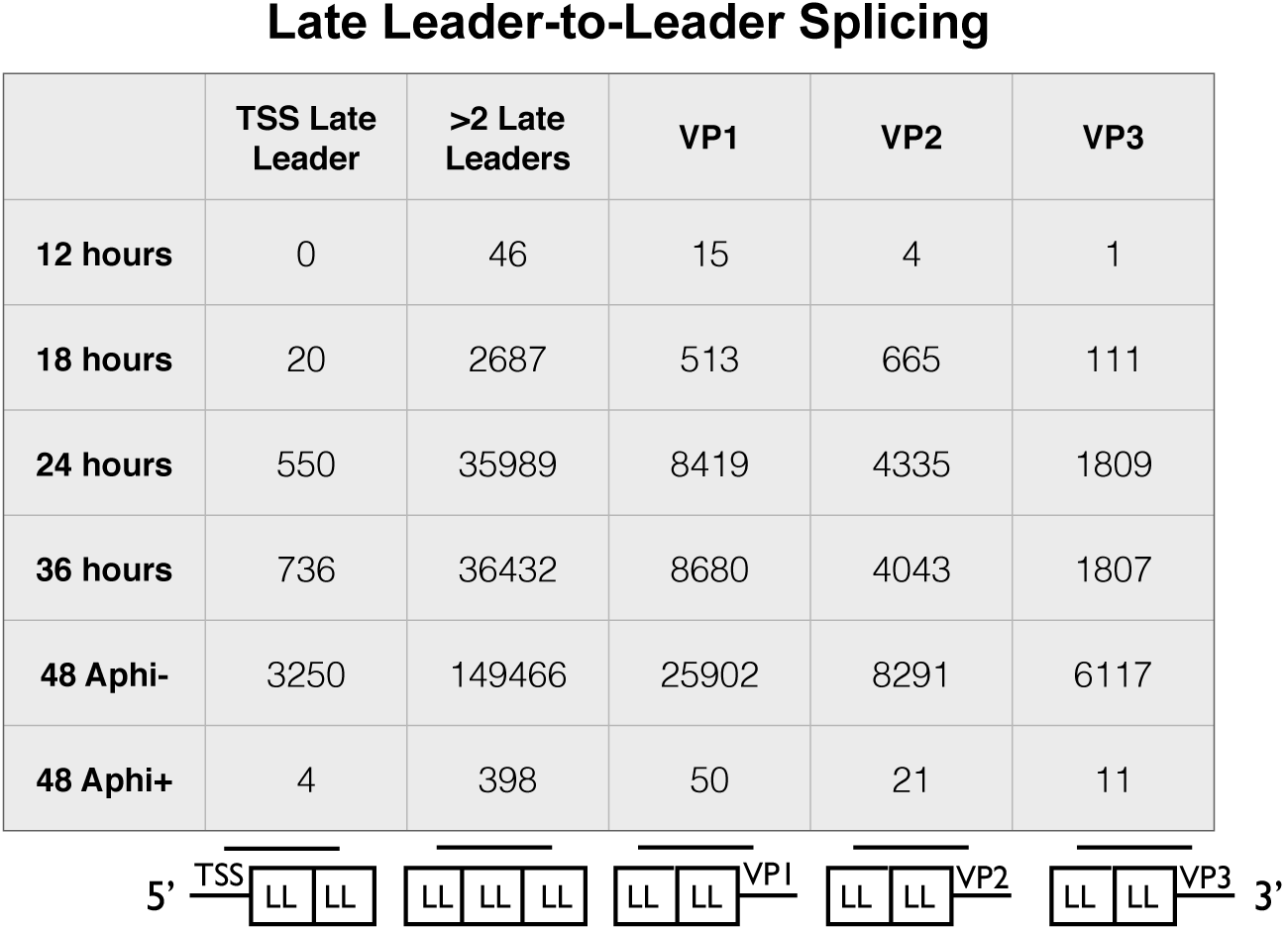
Alignment of reads from late leader repeats. The 100 base reads from time course and aphidicolin samples were realigned to custom reference sequences containing at least one leader to leader splice junction. The reference sequences were 5 variations of a 42-57-42 base splice of late leader exons to each other, the transcription start site, or the 5’UTR of VP1-3. The number of reads aligning to each reference sequence for each condition are shown.

Owing to the presence of tandem repeats of late leaders in all three late mRNAs, alternative reference sequences were required to capture reads that span at least one leader-to-leader splice junction (Fig 4). The stranded cDNA libraries produce 2x100 base pair reads. The late leader exon is 57 bases long so we required a reference sequence that captured reads that extended at least 1 base past the first leader-to-leader splice junction both downstream and upstream of the late leader on VP1, VP2, and VP3 cDNA as well as the late transcription start sites. We used a reference sequence containing 42 bases upstream of the late leader exon near the transcription start region followed by a full late leader exon and the first 42 bases of the next late leader (Fig 4 bottom of first column). We also used a reference sequence of the last 42 bases of late leader, a full late leader, and the first 42 bases of late leader to capture ambiguous leader to leader reads from any mRNA crossing at least 2 leader to leader splice junctions (Fig 4 bottom of second column). Reference sequences containing the last 42 bases of a late leader followed by a full late leader and 42 bases of the 5’ UTR of VP1, VP2, or VP3 (Fig 4 bottom of third, fourth, and fifth columns). Leader-to-leader splices were only seen at significant levels in time points later than 18 hours and in cells untreated with aphidicolin, supporting the model that leader to leader splicing occurs after DNA replication. These results are consistent with RT-PCR analyses (S5 Fig).

### A-to-I editing of viral RNAs

Due to the read-through of early and late transcripts at late times, as well as the genomic overlap of the early and late polyadenylation signals, there is the possibility that if complementary sequences accumulate near one another in the nucleus, they might form double stranded RNAs. As nuclear dsRNAs can be promiscuously edited by ADAR enzymes, which deaminate adenosines to inosines [52], viral dsRNAs should also exhibit this fate. We have reported previously that this is indeed the case [46,51], but the current study allowed us to examine viral RNA editing in more comprehensive detail, which was done in two ways. Since inosine base pairs in the same manner as guanosine, we first looked for individual A-G mismatches in the read alignments to search for potential sites for editing. We divided the 100 nucleotide reads into four 25-nucleotide fragments and allowed up to three base mismatches during the alignment step [63]. After filtering for any sequences that aligned to the mouse genome, we realigned the sequences to the viral genome to show regions of A-G mismatches (S6 Fig). Such alignment cannot be conclusive for RNA editing, as it could merely reflect sequencing or alignment errors. Indeed, this sort of analysis, when applied to other base transformations, generated similar, though less robust, data (S2 Table). We therefore examined viral regions where A-G mismatches greatly outnumbered mismatches caused by any other base changes. The most striking region associated with A-G changes was the overlapping polyadenylation region, which showed a much higher rate of A-G mismatch compared to other combinations (S2 Table). This was not surprising since the polyadenylation region has previously shown to be edited at late times during infection [46].

**Fig 5:**
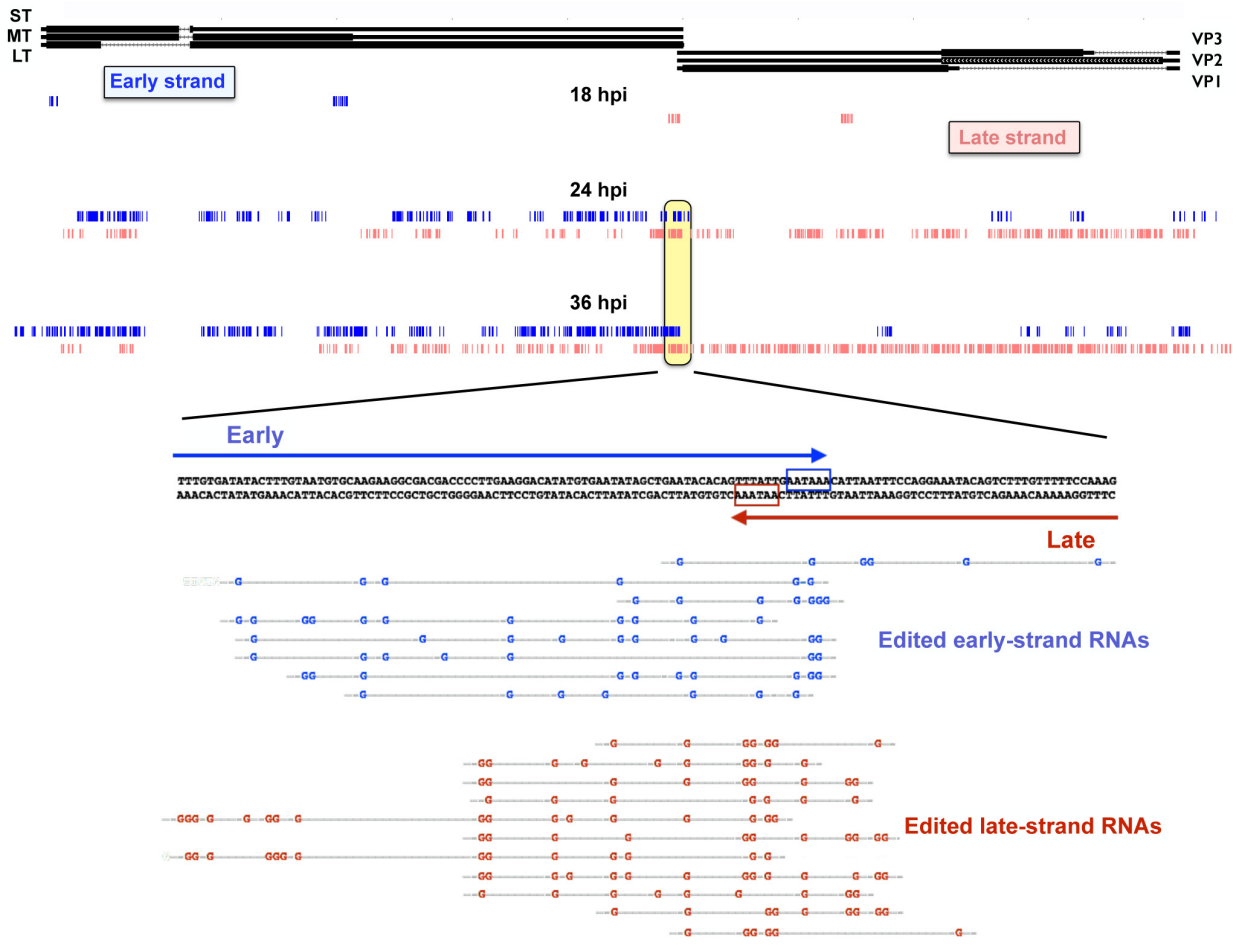
Accumulation of hyper-editing clusters during the time course. Reads from the time course were aligned with the host mouse genome and discarded. Reads were then aligned to the Py59RA reference genome and discarded. As in reads that did not align to the Py59RA genome were changed to Gs and realigned to a Py59RA reference genome that itself had all As changed to Gs. This allowed for reads that did not originally align to the unaltered Py59RA genome due to hyperediting to be captured. The process was repeated for all combinations of single base mismatch. Clusters of A-G mismatches from the time course consistent with hyperediting by ADAR were mapped to the Py59RA genome.

As long dsRNA is subject to hyper-editing, where up to 50% of the adenosines are converted to inosines [51], it is quite likely that the above analysis missed much or most of the editing that occurs during MPyV infection. In order to capture a greater fraction of hyper-edited reads that may have been omitted due to having too many A-G mismatches we used a method recently described by Porath et al [64]. These authors developed a pipeline that sorts unaligned reads based on whether they fail to align due to a specific type of mismatch and whether such mismatches occur in the types of clusters associated with hyper-editing. The first step in the pipeline is to align all the reads to the mouse genome to reduce the total number of reads and remove most non-viral sequences. Second, is to align the remaining reads to the viral genome and discard any that align perfectly or with few mismatches. Third, is to transform all As in both the reads and the reference genome to Gs and discard any transcripts that do not align. This allows us to only sort for reads that initially had A to G mismatches. Fourth, the mismatches are clustered based on predictions for known A-I editing including adjacent sequences and only reads that cluster in a manner consistent with A-I editing are kept. Finally the bases that are members of clusters are displayed on the UCSC genome browser (Fig 5). Results showed a time-dependent increase in the number of bases that were members of hyperedited clusters, with a large concentration of such clusters around the overlapping polyadenylation sites. No significant editing was detected in aphidicolin treated samples. Together, these data are consistent with our previously reported MPyV editing results [46,50]. Shortly after the early late switch at 18 hours we see the polyadenylation region (and a cryptic poly(A) site in the early region, just downstream of the stop codon for middle T antigen) showing editing clusters. As the infection progresses the number and size of the clusters increases on both strands. This is consistent with editing resulting from early and late strand hybridization since the over abundance of late sequence compared to early would only produce more clusters if the late strand formed hairpins and was edited in *cis*. Editing clusters can also be seen in the noncoding control region suggesting that multi-genome length giant transcripts are capable of annealing with one another. While editing has long been predicted due to the long antisense transcripts and poly(A) read-through, this is the first time we have had direct evidence of genomewide editing at late times. We note, however, that while editing is readily and abundantly observed, we cannot conclude from these data whether it is a cause or a consequence of viral gene regulation.

### Effects of viral infection on host gene expression

Our deep sequencing data allowed us to not only examine viral gene expression during infection, but also changes in the host transcriptome that result from the infection. Using Cuffdiff to compare gene expression between MPyV and mock infected samples, we found 1,216 genes were differentially expressed at least 1.5 fold across all three replicates with 359 genes upregulated and 857 genes downregulated compared to the mock infections at the 36 hour time point (Tables 2, S3 and S4).

**Table 2:** Number of host genes differentially expressed between mock and Py59RA infected samples.

	12 hours	18 hours	24 hours	36 hours
Upregulated	0	6	12	359
Downregulated	0	7	19	857
Total	0	13	31	1,217

The number of host transcripts that changed more than 1.5 fold across all three biological replicates was determined using Cuffdiff. Only genes with expression >1 fpkm were counted.

We used the database for annotation, visualization, and integrated discovery (DAVID) (http://david.abcc.ncifcrf.gov/) to determine the gene ontology of the differentially expressed genes that were significantly enriched based on common biological function (Table 3). We also selected genes of high abundance with a strong up or down regulation between mock and virus infected cells at 36 hours for validation by RT-qPCR (S7 and S8 Figs).

**Table 3:** Gene ontology analysis.

	Gene Count	% of upregulated genes	P-value	Fold Enrichment	FDR
**Upregulated**					
Ribosomal Protein	38	11.95	3.6E-30	12.86	4.65E-27
RNA Processing	31	9.75	3.29E-10	3.9	5.19E-07
Chromosomal Protein	19	8.5	1.19E-11	8.37	1.53E-08
Mitochondrial Protein	42	13.2	9.3E-11	3.18	1.2E-07
DNA Replication	18	5.66	2.16E-09	6.51	3.41E-06
Ribosome Biogenesis	14	4.4	1.13E-07	6.87	1.79E-04
Pyrimidine Metabolism	16	5.03	1.5E-09	7.59	1.55E-06
**Downregulated**					
GTPase Regulator	58	6.98	1.19E-16	3.49	1.67E-13
Cell Adhesion	61	7.34	8.6E-10	2.35	1.52E-06
Actin Cytoskeleton	32	3.85	7.04E-10	3.66	9.73E-07
Chromosome Organization	44	5.29	2.7E-07	2.36	4.76E-04
Embryonic Development	48	5.77	1.7E-08	2.47	2.99E-05
Transcription Regulation	158	19.01	8.99E-09	1.53	1.59E-05
Urogenital Development	27	3.25	2.6E-09	4	4.59E-06
Cell Motion	44	5.29	1.73E-08	2.59	3.05E-05
Extracellular Matrix	39	4.7	3.69E-09	2.96	5.09E-06
Pathways in Cancer	42	5.05	5.53E-10	2.9	6.27E-07
Zinc Finger	93	11.2	4.9E-08	1.79	6.79E-05

The web based application Database for Annotation, Visualization and Integrated Discovery (DAVID) was used to sort lists of upregulated or downregulated genes by common function and enrichment using the *Mus musculus* background. A false discovery threshold of 1E-03 was used.

**Fig 6:**
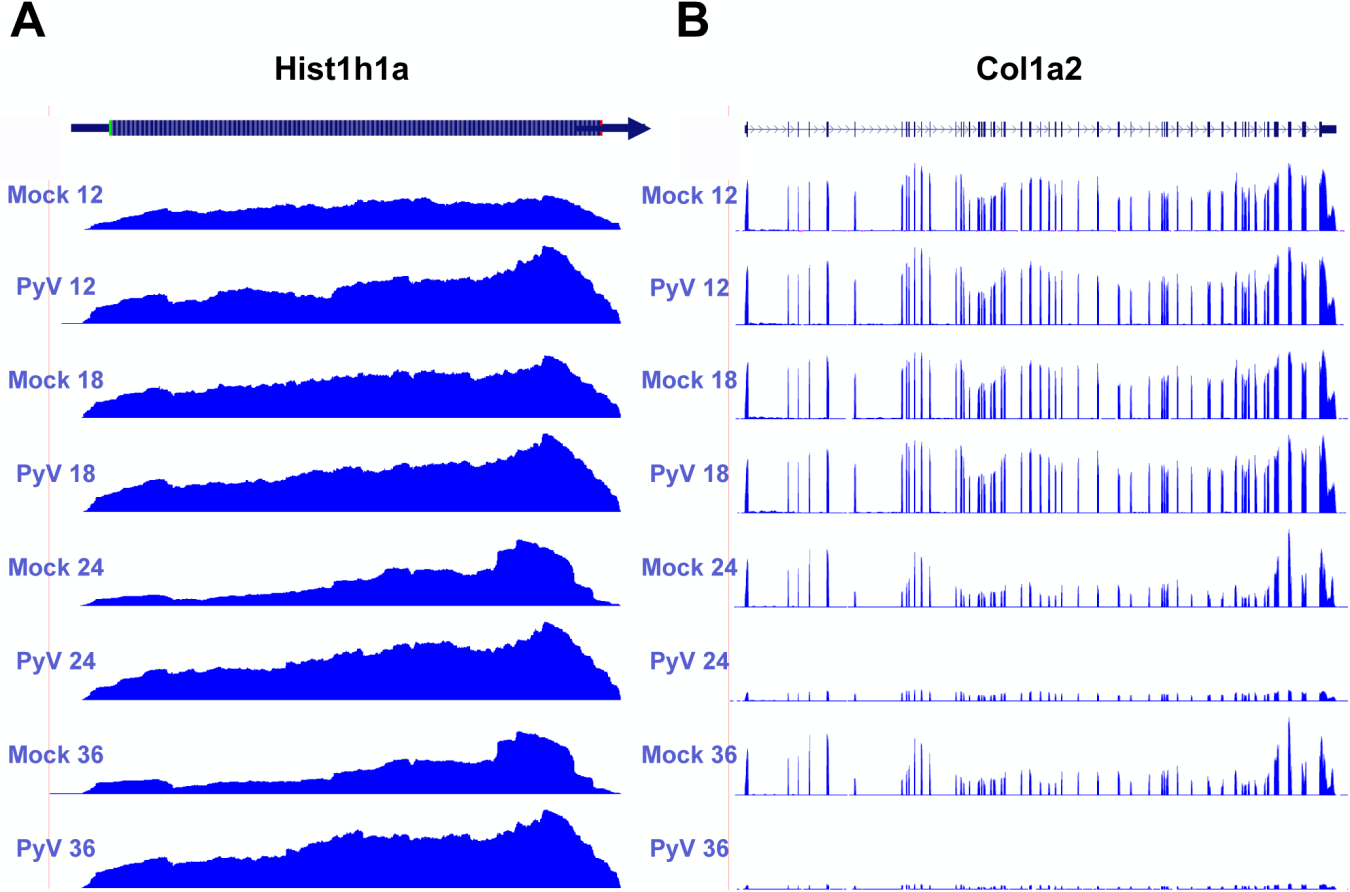
Example of host protein coding transcripts significantly upregulated or downregulated in infected samples compared to mock infection. Protein coding reads from the time course were aligned to the host genome. **(A)** Expression of Hist1ha increases compared to mock infection. **(B)** Col1a2 decreases compared to mock infection.

For the upregulated genes, the categories with the highest enrichment included transcripts coding for ribosomal proteins, chromatin binding proteins, DNA replication, RNA processing, and mitochondrial proteins. These are all consistent with the known increase in growth and metabolism induced by the combined action of the early genes. For example, the transcript for Hist1h1a that codes for the histone 1 linker involved in compacting DNA during replication [65] is upregulated more than 2 fold in infected cells by 36 hours (Fig 6A). The transcript for the mitochondrial inner membrane protein Timm8a1 that is involved in the insertion of cytoplasmic proteins into the mitochondrial inner membrane [66] is also upregulated. Another interesting observation is the transcript coding for the JunB component of AP-1 transcription factors is upregulated. This is of interest because JunB was originally understood to function as an antagonist of the AP-1 protein c-Jun, which is involved in cell cycle progression [67,68]. Later observations found this interaction to be more complex and sensitive to cell type and context [69].

Downregulated genes were enriched in categories such as GTPase regulation, cell adhesion, actin cytoskeleton, extracellular matrix, and transcription regulation. One of the more strongly downregulated transcripts included Col1a2 (Fig 6B) which codes for one of the polypeptide components of type 1 that makes up the extracellular matrix [70]. Another strongly downregulated transcript encodes the steroyl-CoA desaturase 2 (Scd2) enzyme that catalyzes the synthesis of monounsaturated fatty acids, increasing membrane fluidity [71]. Unlike in MPyV infection, Scd2 has been shown to be upregulated in other cancer cells and may be responsible for inducing hepatocellular carcinomas [72]. It is unclear how the virus would benefit from a reduction in monounsaturated fatty acid synthesis. The transcript for the protein myristolylated alanine-rich C-kinase substrate (Marcks) that is involved in altering the actin cytoskeleton [73], cell motility [74], and functions as a tumor suppressor [75] was originally shown to be downregulated in mouse 3T3 fibroblast cells expressing v-Src [76]. Our data also show significant down regulation of this transcript.

**Fig 7:**
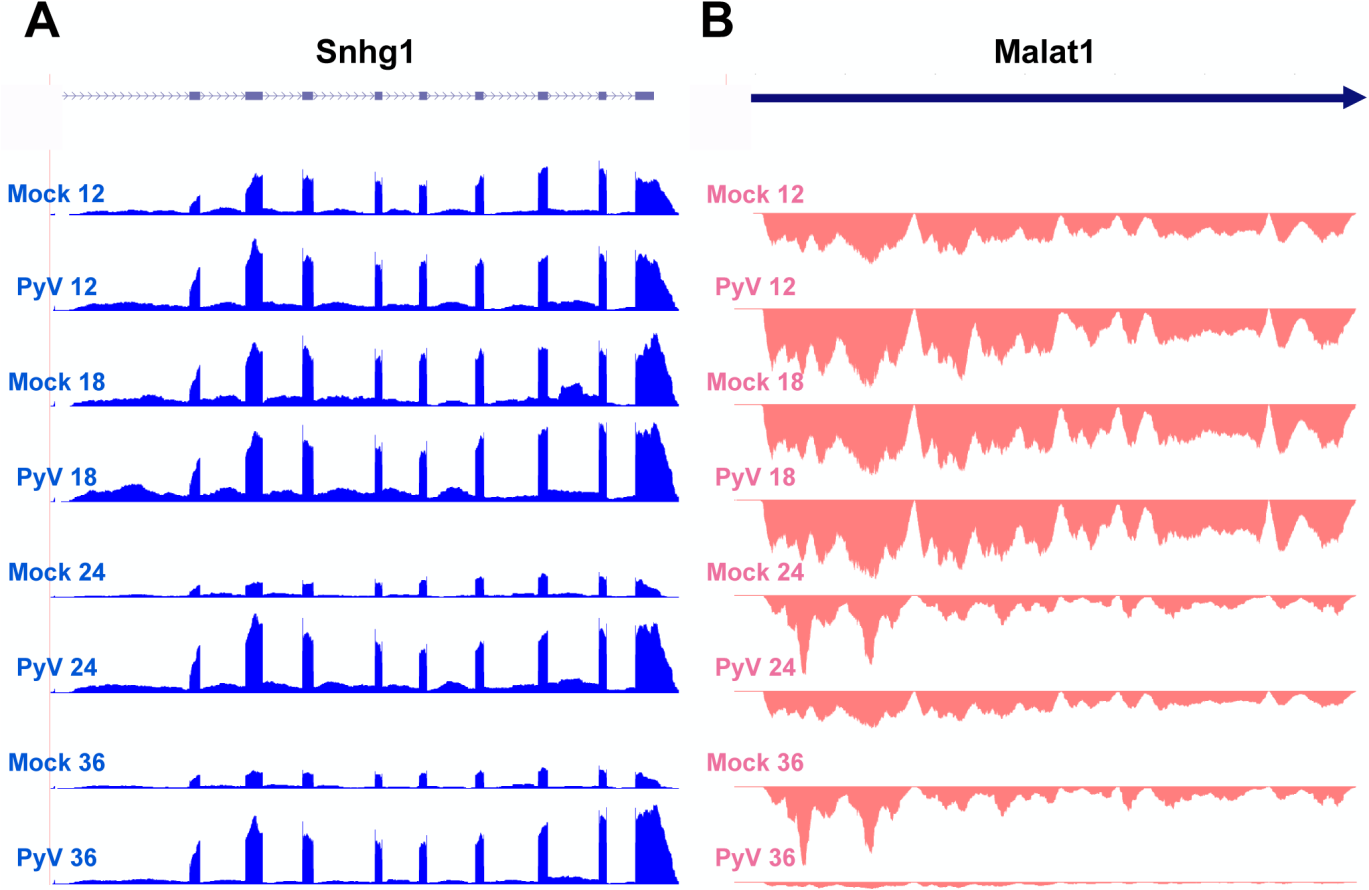
Example of host noncoding transcripts significantly upregulated or downregulated in infected samples compared to mock infection. Noncoding RNA reads from the time course were aligned to the host genome. **(A)** Expression of Snhg2 increases compared to mock infection. **(B)** Malat1 decreases compared to mock infection.

In addition to coding RNAs, we observed significant changes in the expression of noncoding RNAs (S7 and S8 Figs and S4 Table). The noncoding small nucleolar RNA Snhg1 was highly expressed and significantly upregulated (Fig 7A). Snhg1, also known as the host gene for U22, is expressed from an intron and is required for processing of 18S ribosomal RNA [77]. This is consistent with the increase in the expression of genes involved in ribosome biogenesis and translation in MPyV infected cells. Another noncoding RNA, Metastasis Associated Lung Adenocarcinma Transcript 1 (MALAT1), was significantly downregulated in infected cells (Fig 7B). Though the precise function of MALAT1 is still unclear, it has been shown to be involved in a variety of metastatic cancers [78]. Its downregulation is consistent with the downregulation of other genes involved in cell motility in MPyV-infected cells.

Taken together, these observations illustrate a change in priority of infected cells from cell-to-cell interaction, movement, and signal transduction to growth, metabolism, and DNA synthesis.

Do differentially expressed genes share common patterns of regulation that may help to explain their response to viral infection? In order to determine common transcription factor binding sites upstream of differentially expressed genes we used the Transfind tool (http://transfind.sys-bio.net/) to determine common transcription factor binding sites among the differentially expressed genes [79]. Using a threshold of binding motifs common to the upstream regions of <1000 genes (out of 33,837 genes in the mouse database) and a false discovery rate <0.02, we found a number of common transcription factor binding sites (Table 4). As expected the promoters of upregulated genes were the most enriched for binding sites of the transcription factor E2F, which is a major cell cycle regulator that is released and activated by the binding of Large T antigen to pRB [80]. Binding sites for members of the CREB-ATF family of transcription factors were also enriched in the upregulated gene set. This is consistent with CREB Binding Protein (CBP) and EP300 being targets for Large T antigen, resulting in CRE containing promoters being activated [24]. The nuclear factor kappa B (NFkB), which has been previously shown to be involved in inflammation, transformation, proliferation, angiogenesis, and metastasis shows binding sites upstream of the upregulated genes [81]. Interestingly, STAT1, which is involved in signal transduction of the interferon pathway, appears to have a common binding site among a number of the upregulated genes. While the interferon pathway does not appear to be active (pathway genes were not noted to be strongly up or down regulated during infection), the fact that many upregulated genes share the potential to be affected by it is of interest.

**Table 4:** Transfind transcription factor prediction.

**36 hour upregulated**							
**Factor**	**p-value**	**FDR**	**FP**	**# of genes +**	**# of genes -**	**Background +**	**Background -**
E2F	<1.00E-07	3.000E-09	9.000E-09	45	331	955	32882
NF-Y	2.74E-07	1.486E-05	5.944E-05	31	345	969	32868
HIF1	8.45E-07	3.057E-05	1.834E-04	30	346	970	32867
GABP	2.517E-06	6.827E-05	5.462E-04	29	347	971	32866
CREBATF	2.517E-06	6.827E-05	5.462E-04	29	347	971	32866
STAT1	1.999E-05	4.338E-04	4.338E-03	27	349	973	32864
CREB	1.999E-05	4.338E-04	4.338E-03	27	349	973	32864
CREBP1	5.322E-05	8.249E-04	1.155E-02	26	350	974	32863
ATF4	5.322E-05	8.249E-04	1.155E-02	26	350	974	32863
AHR	5.322E-05	8.249E-04	1.155E-02	26	350	974	32863
NFkB	7.882E-04	1.069E-02	1.710E-01	23	353	977	32860
YY1	7.882E-04	1.069E-02	1.710E-01	23	353	977	32860
ATF2CJUN	1.778E-03	1.930E-02	3.859E-01	22	354	978	32859
C-REL	1.778E-03	1.930E-02	3.859E-01	22	354	978	32859
IRF1	1.778E-03	1.930E-02	3.859E-01	22	354	978	32859
ATF3	1.778E-03	1.930E-02	3.859E-01	22	354	978	32859
**36 hour downregulated**							
SP1	<1.00E-09	3.000E-09	2.600E-08	85	781	915	32432
WT1	<1.00E-09	3.000E-09	2.600E-08	82	784	918	32429
AP2	<1.00E-09	3.000E-09	9.000E-09	81	785	919	32428
TFIII	<1.00E-09	3.000E-09	2.600E-08	70	796	930	32417
MAZ	<1.00E-09	3.000E-09	2.600E-08	69	797	931	32416
HIC1	<1.00E-09	3.000E-09	2.600E-08	66	800	934	32413
KROX	<1.00E-09	3.000E-09	9.000E-09	64	802	936	32411
AHRHIF	6.000E-09	1.100E-07	1.210E-06	58	808	942	32405
MZF1	3.400E-08	6.150E-07	7.385E-06	56	810	944	32403
MAZR	2.220E-06	3.442E-05	4.818E-04	51	815	949	32398
HES1	2.220E-06	3.442E-05	4.818E-04	51	815	949	32398
SPZ1	4.816E-06	6.967E-05	1.045E-03	50	816	950	32397
EGR1	1.023E-05	1.306E-04	2.219E-03	49	817	951	32396
MTF1	1.023E-05	1.306E-04	2.219E-03	49	817	951	32396
EGR2	2.126E-05	2.428E-04	4.613E-03	48	818	952	32395
ZIC1	2.126E-05	2.428E-04	4.613E-03	48	818	952	32395
ZIC2	4.324E-05	4.691E-04	9.382E-03	47	819	953	32394
AHR	8.601E-05	8.483E-04	1.866E-02	46	820	954	32393
USF	8.601E-05	8.483E-04	1.866E-02	46	820	954	32393
CACCCBF	1.673E-04	1.578E-03	3.630E-02	45	821	955	32392
HIF1	3.181E-04	2.761E-03	6.902E-02	44	822	956	32391
SMAD4	3.181E-04	2.761E-03	6.902E-02	44	822	956	32391
PAX3	1.072E-03	8.310E-03	2.327E-01	42	824	958	32389
NRF1	1.072E-03	8.310E-03	2.327E-01	42	824	958	32389
AP2a	1.072E-03	8.310E-03	2.327E-01	42	824	958	32389
USF	1.900E-03	1.422E-02	4.123E-01	41	825	959	32388

The web based application Transfind was used to predict common transcription factor binding sites among the lists of upregulated and downregulated host genes. Predictions of mouse transcription factor binding sites were made with promoter sets of 1000nt from 800nt upstream to 200nt downstream and compared with 1000 genes with the highest predicted factor affinities among 33,837 total mouse genes. Possible factors were represented by the TRANSFAC matrix.

## Discussion

We have presented here a global view of MPyV infection with respect to both the virus and the host transcriptomes. The data and analyses have allowed us to generate a detailed picture of how viral gene expression changes throughout the life cycle as well as to learn how infection impacts host cell gene expression. We analyzed RNA from a number of individual time points, from relatively early in infection (12 hours post infection) to late times (36 hours post infection) but before extensive cytopathic effects were noted. Although there were several surprises, the observed expression and processing of viral transcripts was generally consistent with previous literature. At early times in infection, early-strand transcripts dominated over late-strand transcripts. This early pattern is even more dramatic when DNA replication is blocked with aphidicolin and the infection allowed to proceed for 48 hours. At early times, most of these transcripts appear to initiate at a defined site and terminate just downstream of the annotated early polyadenylation signal. These are spliced to produce mRNAs for the three viral early proteins, Large, middle and small T antigens. However, by 18 hours, after Large T antigen has promoted the onset of viral DNA replication, the pattern of expression reveals a striking shift toward the late phase, where late-strand RNAs greatly dominate over early-strand RNAs. This early-to-late switch is characterized by several distinct and important changes in the pattern of alignments in our data. First, as late messages accumulate rapidly and genomes replicate, we noted an unexpected shift in the position of the 5’-ends of early-strand transcripts. At late times, early-strand RNAs appear to initiate over a broad region upstream of the early TATA box, and throughout the origin of replication and enhancer region. We do not yet know why this is the case or how it is regulated, but these findings are consistent with an earlier reports in which heterogeneous 5’-ends at late times were mapped using nuclease S1 protection [58,59] as well as with studies on both SV40 and JC virus [60,61]. Interestingly, this shift depends on viral DNA replication, since after blocking it with aphidicolin, these upstream 5’-ends were not seen, even after 48 hours. One possible explanation for this observation is the fact that the A enhancer on the late side of the origin also functions as a bidirectional promoter site for the late transcripts [82]. Since this shift in early transcription is only seen in the late phase, it is possible this region is transcribing bidirectionally. We note that since large T antigen is expressed during aphidicolin treatment, this shift also depends on replication and not just on viral early proteins. Further studies are needed to determine the molecular mechanism for this phenomenon.

Another clear change in early-strand expression, and also dependent on DNA replication, was the appearance and accumulation of reads mapping to a region several hundred nucleotides downstream of the major early polyadenylation site. These reads appear to result from poly(A) site read-through and are antisense to VP1. While we do not yet know why or how they accumulate, we considered one possible explanation to be that they represent precursors for viral-encoded microRNA(s). MPyV and SV40 have previously shown viral expression of microRNAs antisense to Large T antigen, which were hypothesized to help avoid a cytotoxic T cell response [83,84]. Upon analysis of the sequence in the region, we found no predictors of stem loop structures on either strand that would be consistent with a viral premiRNA. Alternatively, the increase in sequences from this region could result from uneven processing/degradation of early-strand transcripts that have read through the early polyadenylation site. However, the absence of clearly defined ends of these mapped reads is hard to justify with specific processed products. Importantly, we cannot rule out the possibility that sequences aligning to this region result from an artifact of a bias in the synthesis or processing of the sequencing libraries. Consistent with such a hypothesis, we note that these reads consistently represented about 3% of the level of reads mapping to the complementary late strand in this region of the genome. Also, the early strand in this region contains 15% A’s and 40% T’s. Stranded libraries are made using dUTP incorporation. If complementary transcription of the late transcripts from this region (we are generating about 200 nt cDNAs) inefficiently incorporates dUTP for any reason, then the strandedness would be compromised. In our protocols, this type of artifact may be happening at numerous places, but at much lower levels than in the region in question. For example there is a smaller peak between the early poly(A) site and the peak in question that appears to accumulate in exactly the same way.

Late-strand transcription and processing also changes during infection. While accumulating to only low levels before viral replication, or in its absence, these RNAs accumulate rapidly afterwards, and by late times represent the great majority of viral RNAs in the cell. Their processing may also change with time, although we did not note any discernible change in transcription start sites. As we and others have reported before, late transcription can proceed many times around the circular viral genome, leading to a heterogeneous collection of giant transcripts that can serve as precursors to mature late mRNAs [46–50]. Processing involves not only alternative splicing to generate messages for VP1, VP2 and VP3, but also the splicing of noncoding late leader exons at their 5’-ends. The number of tandem leader units appearing on late mRNAs reflects the number of transits around the genome made by RNA polymerase II before transcription termination and polyadenylation. We noted a striking accumulation of leader-leader splicing events at late times in infection. These were only seen at late times, and after DNA replication.

Late leader splicing requires inefficient late-strand transcription termination and polyadenylation. We have reported previously that this is the result of particular viral genomic features in the early/late polyadenylation region [46]. These sites overlap on the genome and this affords viral transcripts the opportunity to anneal with one another, leading to dsRNA in the nucleus. dsRNA formation could alter normal 3’-end processing by interfering with the polyadenylation machinery. Also, dsRNA formation could lead to promiscuous editing by ADAR1, leading to RNAs that cannot be recognized by the processing machinery. We have previously found that viruses that cannot generate early-strand and late-strand transcripts with regions of overlap are incapable of the early-late switch [46]. The consequences of such dsRNA formation are of profound importance for the virus. Inefficient late-strand termination leads to the multigenomic transcripts that serve as precursors to late mRNAs [47–50]. Early-strand termination is also likely to become less efficient, but in this case the multigenomic transcripts are not used for mRNA production, but are rather likely degraded in the nucleus owing to unproductive splicing. Our data support these concepts. While our transcriptomic data reveal mostly the accumulated mature mRNAs at late times, close inspection reveals roughly equal levels of poly(A) site readthrough transcripts from each strand. These would be expected to be capable of dsRNA formation, leading to ADAR editing. Indeed, A-to-G mismatches consistent with A-to-I editing were observed to only occur at late times (but not in cells untreated with replication inhibitor), with robust editing observed in the overlapping polyadenylation site region. The hyperediting pipeline we employed allowed us for the first time to capture MPyV reads that would otherwise be too heavily altered to be identified. The increase in edited clusters aligning along the entire length of the reference genome was predicted but, to our knowledge, has never been directly observed until now. Taken together, these observations confirm previous models of the early-late switch that describe a loss of polyadenylation efficiency concomitant with an increase in editing of the polyadenylation sites and resulting in giant multigenomic RNAs, which can be spliced to generate multiple late leader exons on late mRNAs.

Alignment of reads from the time course infection to the host genome showed the greatest change between mock and infected samples by 36 hours, allowing us to observe the accumulated effects of the virus on the host transcriptome. We suspect that examination of even later time points might yield more genes changing more than 2 fold up or down, but at time points closer to 72 hours cells would be entering the lysis phase of the viral life cycle and any observed changes would likely be difficult to separate from those associated with host cell death. At 36 hours post infection, we found 359 genes significantly upregulated and 857 genes significantly downregulated. Many of the genes upregulated were involved in predicted processes such as metabolism, ribosome synthesis, mitochondrial proteins, and cell cycle progression. This is consistent with the action of viral T antigens in stimulating the proliferation of host cells. Many downregulated genes were also largely unsurprising, with those involved in cell motility, extracellular matrix, and cell signaling processes prominent on the list. We note that our results are somewhat consistent with those published by the Wintersberger group, where small t and Large T were individually induced in NIH3T3 cells, followed by microarray analysis [85]. Those studies showed that overexpression of each of these proteins for 28 hours (middle T was not studied) led to numerous up or down changes, most in the 2-fold range. Our system is different in that it follows a natural infection (and in different cells, which may already be partially transformed) which expresses not individual viral proteins, but all of them at the same time.

Finally, we noted the upregulation or downregulation of several abundant long noncoding RNAs. There are several lncRNAs of particular interest. One is Malat1 (downregulated), which has been associated with metastasis and which may play a role in RNA processing [76]. Another is Terc (upregulated), which is involved in telomere maintenance and cellular lifespan [86]. Finally, several lncRNAs associated with RNAse P enzymes are also strongly upregulated. We do not yet know the significance of this, but such molecules have been implicated in tRNA and ribosomal biogenesis, which is consistent with the observed increase in host translation in infected samples [87].

Here we have presented for the first time a global analysis of both viral and host transcriptional changes from a mouse polyomavirus infection. The viral transcriptome analysis revealed insights into changes in RNA expression, especially with respect to the early strand at late times and confirmed a number of previous observations of the MPyV lifecycle. This is the most direct evidence of widespread hyper-editing of viral RNA predicted from a model of extensive early and late strand transcription. The observations of changes in host transcription also confirm a number of predictions including the increase in genes involved in cell growth, metabolism, and translation. Future investigation into more precise aspects of the lifecycle including later time points, nuclear and cytoplasmic fractionation, and poly(A) plus and minus samples may yield further insight into the viral-host interactions.

## Materials and Methods

### Cell culture and virus infection

NIH 3T6 mouse fibroblast cells were cultured on 10 cm plates at a density of 600Kcells/plate in Gibco Dulbecco’s Modified Eagle Medium (11995–065) supplemented with 100 units of penicillin/streptomycin, 2mM L-Glutamine, and 5% calf serum. Cells were serum starved in 2% calf serum 1 day before infection to synchronize cell cycles. Mouse polyomavirus strain Py59RA was generously provided by Dr. Robert Garcea in 2% Medium at 180 million plaque forming units per ml. In our hands, higher apparent multiplicities were required to ensure maximal infection of our cells in culture. Virus was diluted to 15 million PFU/ml to infect cells at an MOI of 50 using 2 ml of virus per plate. Cells were infected by removing media and adding 2 ml of virus for 2 hours before being supplemented with 8ml of 2% media at time point 0. Mock infections were carried out using 2% medium without filtered virus particles. Total RNA was harvested at 12, 18, 24, and 36 hours after infection by removing media, washing with PBS, and adding 4M guanidinium isothiocyanate. For the aphidicolin treated samples, cells were plated at the same density, treated with the same media, and infected at the same MOI for 40 and 48 hours. Aphidicolin positive cells were treated with media containing 2μg/ml aphidicolin.

### Preparation of TruSeq stranded library

RNA was isolated by cesium centrifugation through 2 ml of 5.7M cesium chloride at 39000 rpm at 20°C for 18 hours and DNAse treated with DNAse 1 from the Ambion DNAse I Kit. 1 μg of RNA was used from each sample for library preparation with the TrueSeq Stranded Total RNA kit from Illumina. Ribosomal RNA was depleted using rRNA binding beads and the RNA was fragmented and primed with random hexamers. First strand synthesis was done using a mixture containing Superscript II and Actinomycin D to synthesize RNA dependent DNA and inhibit DNA dependent RNA respectively. Second strand synthesis was done using dUTP instead of dTTP to create stranded cDNA. Libraries were then adenylated at the 3’ end and adaptors containing identifier sequences and flow-cell binding sequences were ligated to both ends. Finally the cDNA fragments were amplified for 12 cycles instead of 15. The Agilent Technologies 2200 Tapestation was used to verify library size at ~260bp. Sequencing was done on a HiSeq 2000 by Perkin Elmer.

### Analysis

All alignments were made using Tophat v 2.0.9, Bowtie 2.1.0, and Samtools 1.19. Alignments to MPyV were done using the reference genome for the Py59RA strain of mouse polyomavirus for both time course and aphidicolin experiments. Time course experiments consisted of four conditions, for mock infection and four conditions for MPyV infection representing 12, 18, 24, and 36 hour time points with three biological replicates per condition. Aphidicolin experiments consisted of two conditions Aphi+ and Aphi- with two biological replicates.

Alignment of time course reads to the host genome was done using the reference genome from Illumina’s IGENOMES collection, which in turn was taken from USCS March 5, 2013. Custom scripts were used to postprocess Tophat output to produce strand-specific bedGraph files suitable for use on the UCSC genome browser.

Alignment of reads to the viral origin was done using a custom script that rotated the genome zero position counterclockwise by 149nt to avoid splitting annotated transcripts. The reads were aligned to the “rotated” reference genome using Tophat with a new Bowtie index and transcriptome index. A custom script was then used to convert the new alignments to the original coordinates and the new alignments were added to the original alignments and displayed on the UCSC genome browser.

Alignment of reads containing tandem repeats of the late leader exon from late gene mRNAs that would not align to the viral genome directly was accomplished by doing a direct Bowtie1 end-to-end alignment to predefined reference sequences allowing for up to 2 mismatches. Three references contained 42 bases of the 5’ UTR of VP1, VP2, or VP3 followed by the 57 base late leader exon and the first 42 bases of the next late leader. One reference contained the last 42 bases of the late leader followed by a full 57 base late leader followed by the first 42 bases of the transcription start region upstream of the late leader exon. The last reference sequence contained a full 57 base late leader flanked by 42 bases of each late leader on either side. This allowed 100-base reads spanning at least one leader-to-leader splice junction that would not align more than once to the same reference sequence to align.

A to G mismatches were identified using custom scripts that split the fastq reads into smaller 25nt fragments and filtered out any that could align to the mouse genome. The remaining fragments were aligned to the Py59RA genome using Bowtie1, allowing for up to 3 mismatches. Another custom postprocessing script was used to interpret Bowtie alignment output format and scan mismatch fields for canonical editing substitutions.

The hyperediting scripts from Porath et al. [64] were downloaded and run in the context of our cluster pipeline. Custom scripts were used to extract information from output files, summarize, and plot as tracks on the UCSC genome browser.

The relative expression of host genes between time course Mock and PyV infected samples was determined using Cuffdiff v 2.1.1. Dispersion method pooled and dispersion method per condition were used to compare individual replicates and all replicates of a given condition. Custom scripts were used to post process/filter Cuffdiff output to produce time-series instances of significant log Fold change. Cluster 3.0 was used to produce a k-means clustering of time course gene expression. Genes with a log2 fold change of +/-0.75 representing a 1.5 fold change with an fpkm greater than or equal to 1 were used for gene ontology and transcription factor prediction.

Gene ontology analysis was done using the browser-based application DAVID (Database for Annotation, Visualization, and Integrated Discovery) (http://david.abcc.ncifcrf.gov/home.jsp). Gene lists of upregulated and down regulated genes were each run against a *Mus musculus* background and gene categories with a false discovery rate below 1E-03 were used with redundant categories removed.

Transcription factor binding prediction was done using the browser-based application Transfind (http://transfind.sys-bio.net/). The same gene lists of up and down regulated mouse genes were used with “800 upstream … 200 downstream of TSS (1000nt, long putative promoters)” for the promoter set category and with “1000” genes under the “highest predicted factor affinities to take” category. The TRANSFAC (highest info content for each factor) setting was used to represent the factors. The data for this paper have been deposited at GEO (GSE69314). http://www.ncbi.nlm.nih.gov/geo/query/acc.cgi?acc=GSE69314

## Supporting Information

S1 Fig Infection of NIH3T6 cells by Py59RA. Mock infected or cells infected with polyoma virus for 24 hours were fixed and stained with antibody for Large T antigen. A multiplicity of 50 of pfu measured by an independent method in the laboratory of R. Garcea was found to be optimal for consistent high efficiency infection of our cells in culture.
(TIF)

S2 Fig Library sizes. Libraries used for sequencing were run on Agilent Technologies 2200 Tape Station to verify library size.
(TIF)

S3 Fig Py59RA reference sequence. 5’ splice sites are indicated in blue. 3’ splice sites are indicated in red. The late leader exon is shown with the black box. The early poly(A) site is shown with a blue box. The complement of the late poly(A) site is shown with a red box.
(TIF)

S4 Fig Mapping of early-strand 5’-ends by 5’-RACE. Cells were infected with Py59RA for either 12 or 36 hours, then total RNA was isolated and subjected to 5’-end analysis using the SMARTer RACE 5’/3’ Kit (Clontech Laboratories, Inc.) according to the vendor’s instructions. A gene specific primer (5’-GCCGGTTCCTCCTAGATTCATTCTC) corresponding to positions 370–394 of the Py59RA genome was used to make 5’ RACE cDNA. cDNA was further amplified using another gene specific nested primer (5’-GCAGTGACTGCTGCTTATATGCCTG) corresponding to positions 257–281 of the viral genome. 25 μl aliquots of the PCR reactions were loaded onto a 6% polyacrylamide gel. Lane 1, markers. Lane 2, no template added to the PCR reaction. Lane 3, mouse heart total RNA was used for cDNA synthesis and RACE. Lane 4, 12 hour mock infection RNA. Lane 5, 36 hour mock infected RNA. Lane 6, 12 hour infection RNA. Lane 7, 36 hour infection RNA. Note: the band of approximately 200 bp denotes startsites in the nt 139–144 region of the virus, reported to represent the major startsites for early-strand transcripts in the absence of DNA replication [50]. At 36 hr post infection, a number of longer bands were observed, representing additional upstream transcriptional startsites consistent with our RNA-Seq data and with an earlier report [61]. The diagram at the bottom illustrates the general locations of the early-strand startsites on the genome.
(TIF)

S5 Fig Late leader-to-leader splicing. **A.** Cells were infected for 12, 24 or 36 hours and RNA isolated and subjected to RT-PCR analysis using primers specific for late leader to VP1 splicing or late leader to VP2 junctions. Bands were resolved by polyacrylamide gel electrophoresis. Note that since leader-VP2 junctions are collinear with viral DNA. The bottom bands in the VP2 lanes are contaminated with signal from residual viral DNA. **B.** Cells were infected in the presence or absence of 10 μg/ml cytosine arabinoside (AraC), which inhibits DNA replication and RNA isolated at 24 hrs post infection. RT-PCR for VP1 spliced to the late leader was carried out using 5’-^32^P-labeled RT primers but otherwise as in panel A. Bands were revealed by autoradiography. Note that in the absence of viral replication (AraC treatment), multiple tandem leaders are less frequent than in the presence of viral replication.
(TIF)

S6 Fig Reads with up to 3 A-G mismatches as potential ADAR editing sites. Reads from the time course run were broken from 100 bases to 25 bases and realigned with a threshold of 3 mismatches allowed per 25 base read. Reads with A-G mismatches were visualized using the UCSC genome browser to indicate regions with potential editing sites. The polyadenylation sites showed the highest number of A-G mismatched reads at late times of infection.
(TIF)

S7 Fig Validation of upregulated genes. One noncoding and three coding genes with a high expression and at least 1.5 fold change were selected for validation of upregulated genes by qPCR. FPKM results shown in left column. qRT-PCR results shown in the right column.
(TIF)

S8 Fig Validation of downregulated genes. One noncoding and three coding genes with a high expression and at least 1.5 fold change were selected for validation of downregulated genes by qPCR. FPKM results shown in left column. qRT-PCR results shown in the right column.
(TIF)

S1 Table Totals for time course reads. Reads from the three replicates of each time point in the time course. Total reads shown in the left column. Number and percentage of reads aligned to the mouse genome shown in the middle column. Number and percentage of reads aligned to the virus genome shown in the right column.
(TIFF)

S2 Table Single base mismatches across the polyoma genome. Excel spreadsheet listing all base to base mismatches (columns) at each position in the MPyV genome (rows) on either strand found in the 25-base read alignments allowing for up to 3 mismatches per read.
(XLSX)

S3 Table Gene fpkm for all time points. Excel spreadsheet listing fpkm for all mouse genes at each time point for each replicate.
(XLSX)

S4 Table Significant differences for all time points. Excel spreadsheet listing significant differentially expressed genes between Mock and PyV infected samples for each time point across all three replicates.
(XLSX)

S5 Table Significant changes in noncoding RNAs. List of noncoding RNAs by name, feature type, and fold change log2 ratio that differs more than 1.5 fold between Mock and PyV infected samples by 36 hours after infection.
(TIFF)
