# Supplementary figures and images for: Global Analysis of Mouse Polyomavirus Infection Reveals Dynamic Regulation of Viral and Host Gene Expression and Promiscuous Viral RNA Editing
Source: PLoS Pathog. 2015 Sep 25;11(9):e1005166. doi: 10.1371/journal.ppat.1005166 (PMC4583464; doi:10.1371/journal.ppat.1005166)

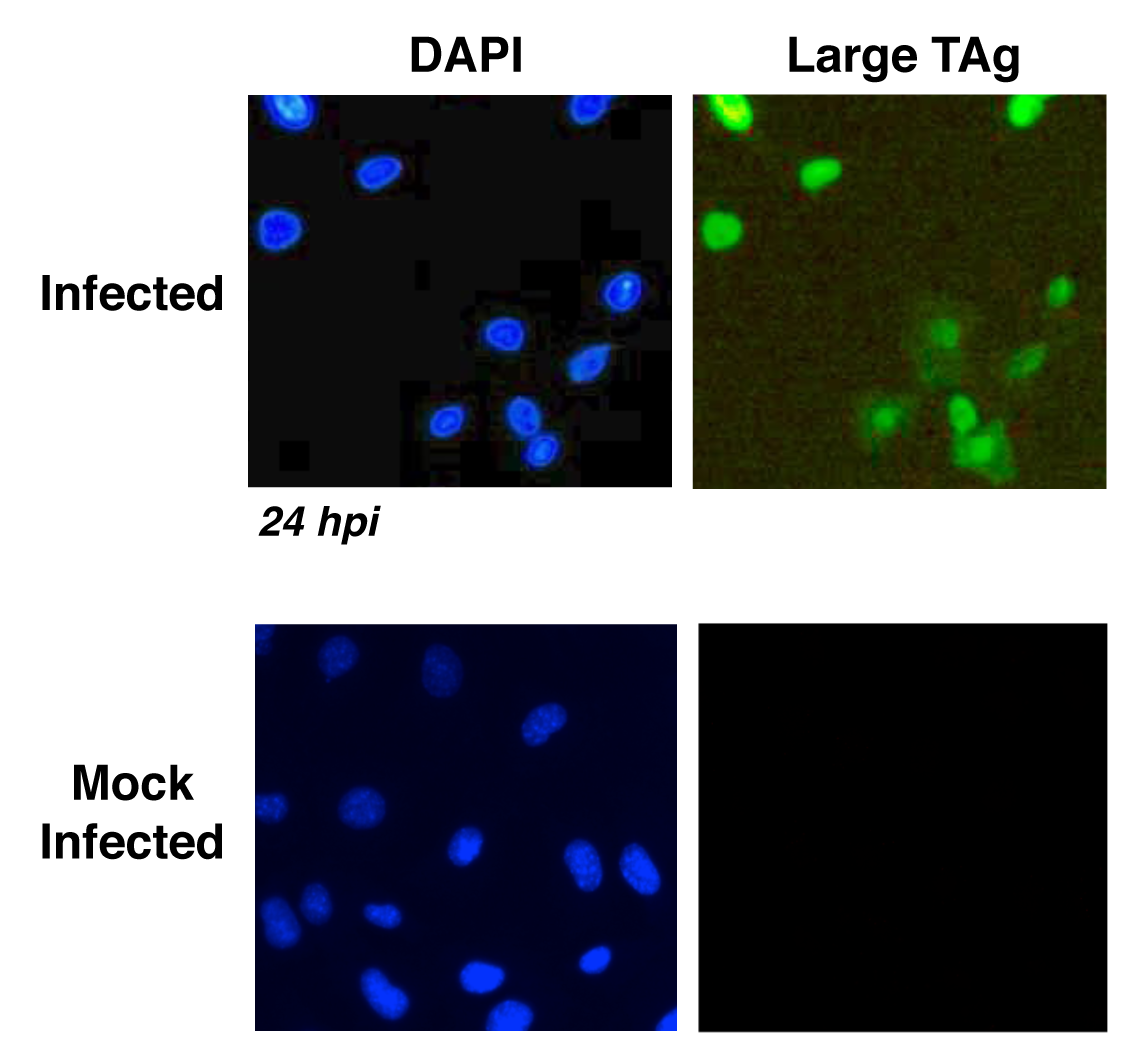

Supplement: S1 Fig — Mock infected or cells infected with polyoma virus for 24 hours were fixed and stained with antibody for Large T antigen. A multiplicity of 50 of pfu measured by an independent method in the laboratory of R. Garcea was found to be optimal for consistent high efficiency infection of our cells in culture. (TIF) [file ppat.1005166.s001.tif]

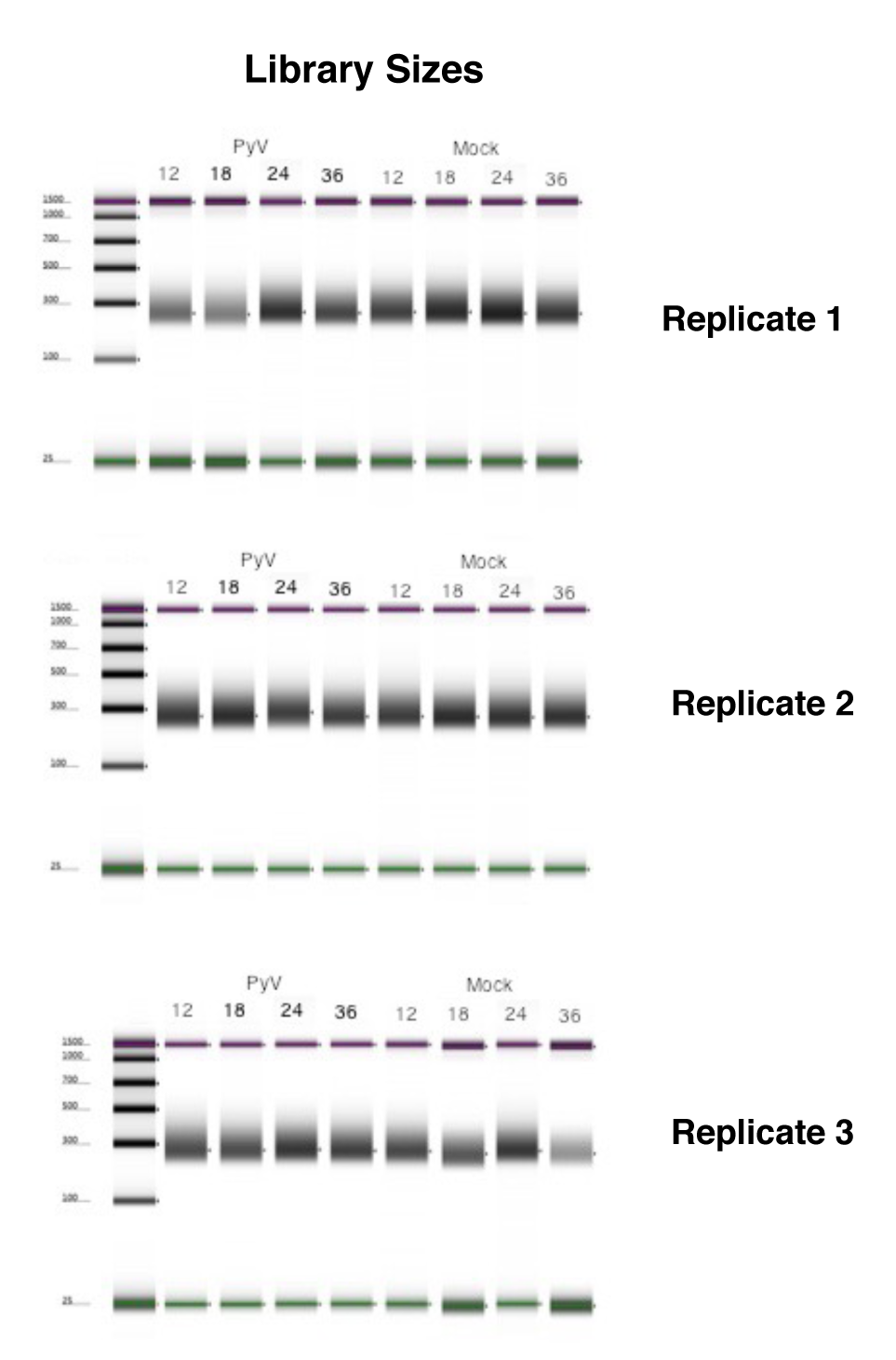

Supplement: S2 Fig — Libraries used for sequencing were run on Agilent Technologies 2200 Tape Station to verify library size. (TIF) [file ppat.1005166.s002.tif]

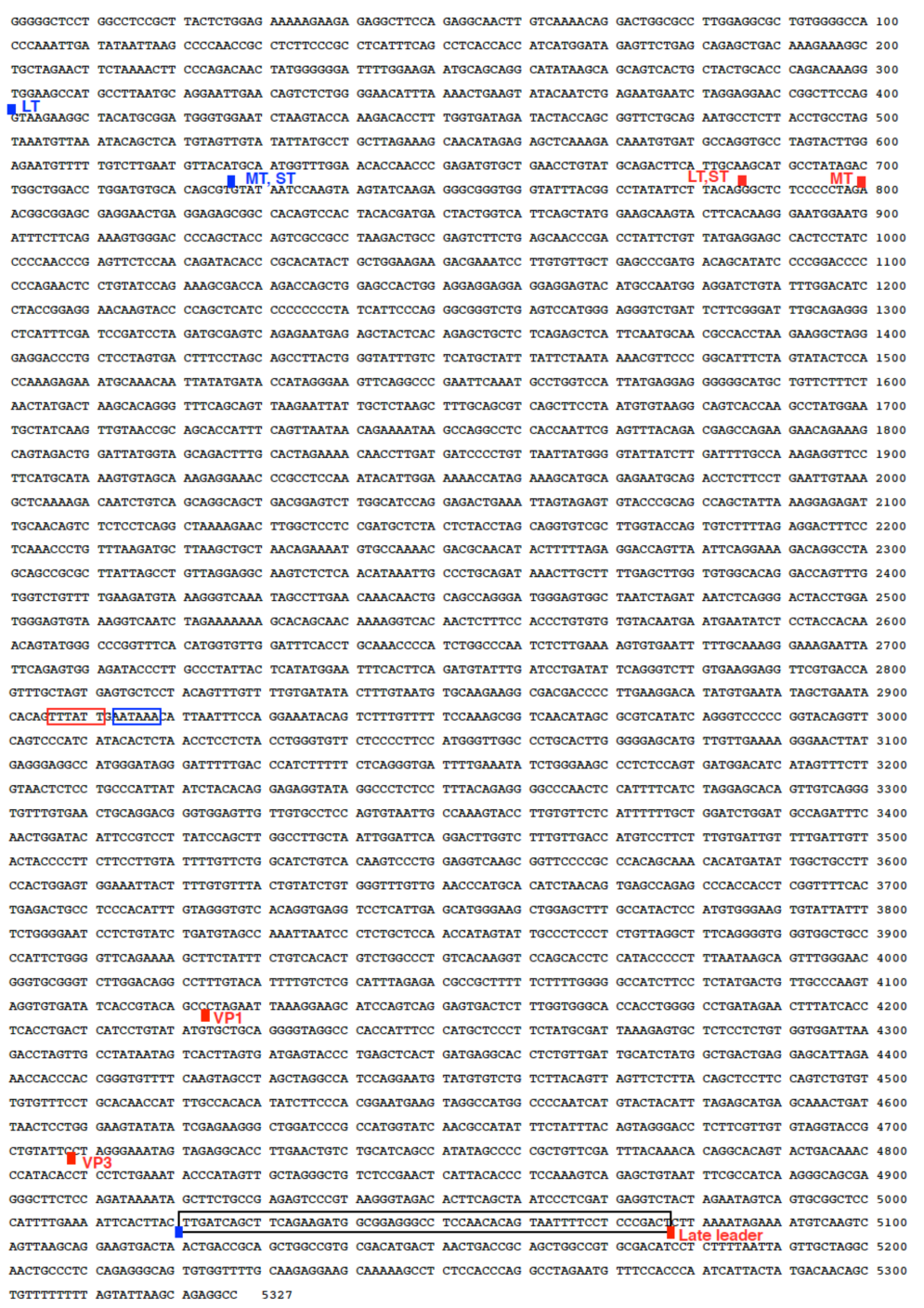

Supplement: S3 Fig — 5’ splice sites are indicated in blue. 3’ splice sites are indicated in red. The late leader exon is shown with the black box. The early poly(A) site is shown with a blue box. The complement of the late poly(A) site is shown with a red box. (TIF) [file ppat.1005166.s003.tif]

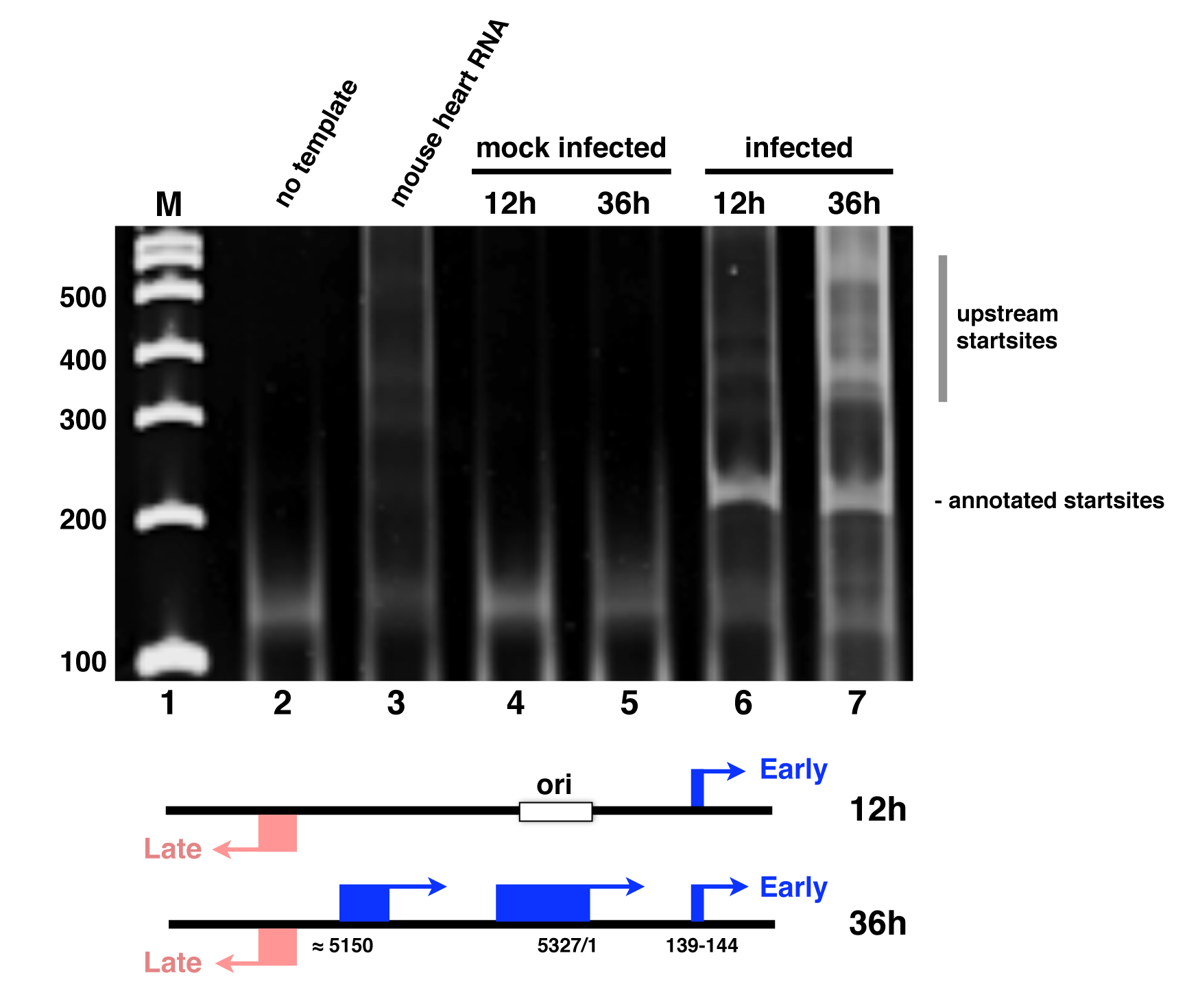

Supplement: S4 Fig — Cells were infected with Py59RA for either 12 or 36 hours, then total RNA was isolated and subjected to 5’-end analysis using the SMARTer RACE 5’/3’ Kit (Clontech Laboratories, Inc.) according to the vendor’s instructions. A gene specific primer (5’-GCCGGTTCCTCCTAGATTCATTCTC) corresponding to positions 370–394 of the Py59RA genome was used to make 5’ RACE cDNA. cDNA was further amplified using another gene specific nested primer (5’-GCAGTGACTGCTGCTTATATGCCTG) corresponding to positions 257–281 of the viral genome. 25 μl aliquots of the PCR reactions were loaded onto a 6% polyacrylamide gel. Lane 1, markers. Lane 2, no template added to the PCR reaction. Lane 3, mouse heart total RNA was used for cDNA synthesis and RACE. Lane 4, 12 hour mock infection RNA. Lane 5, 36 hour mock infected RNA. Lane 6, 12 hour infection RNA. Lane 7, 36 hour infection RNA. Note: the band of approximately 200 bp denotes startsites in the nt 139–144 region of the virus, reported to represent the major startsites for early-strand transcripts in the absence of DNA replication [50]. At 36 hr post infection, a number of longer bands were observed, representing additional upstream transcriptional startsites consistent with our RNA-Seq data and with an earlier report [61]. The diagram at the bottom illustrates the general locations of the early-strand startsites on the genome. (TIF) [file ppat.1005166.s004.tif]

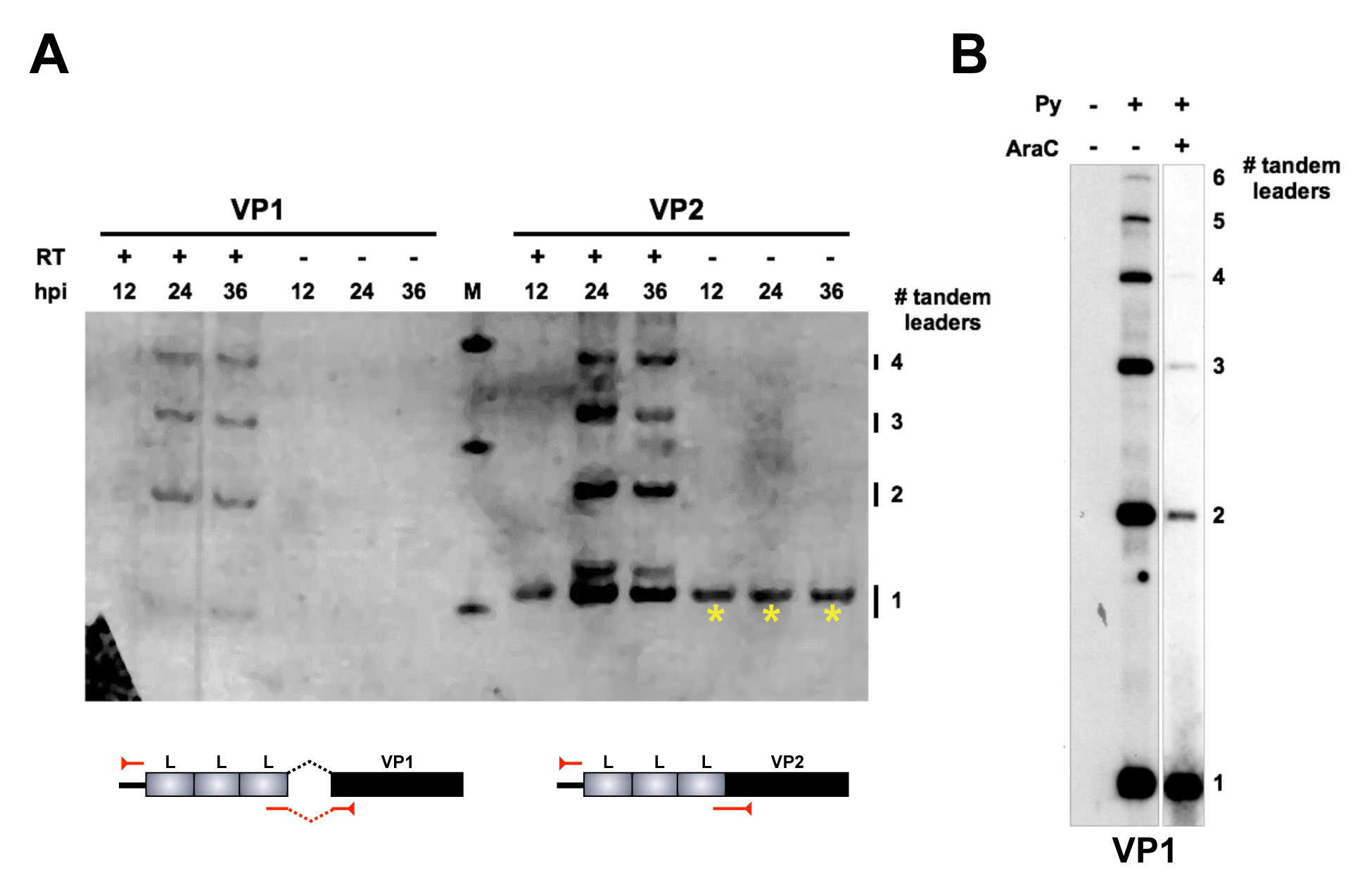

Supplement: S5 Fig — A. Cells were infected for 12, 24 or 36 hours and RNA isolated and subjected to RT-PCR analysis using primers specific for late leader to VP1 splicing or late leader to VP2 junctions. Bands were resolved by polyacrylamide gel electrophoresis. Note that since leader-VP2 junctions are collinear with viral DNA. The bottom bands in the VP2 lanes are contaminated with signal from residual viral DNA. B. Cells were infected in the presence or absence of 10 μg/ml cytosine arabinoside (AraC), which inhibits DNA replication and RNA isolated at 24 hrs post infection. RT-PCR for VP1 spliced to the late leader was carried out using 5’-32P-labeled RT primers but otherwise as in panel A. Bands were revealed by autoradiography. Note that in the absence of viral replication (AraC treatment), multiple tandem leaders are less frequent than in the presence of viral replication. (TIF) [file ppat.1005166.s005.tif]

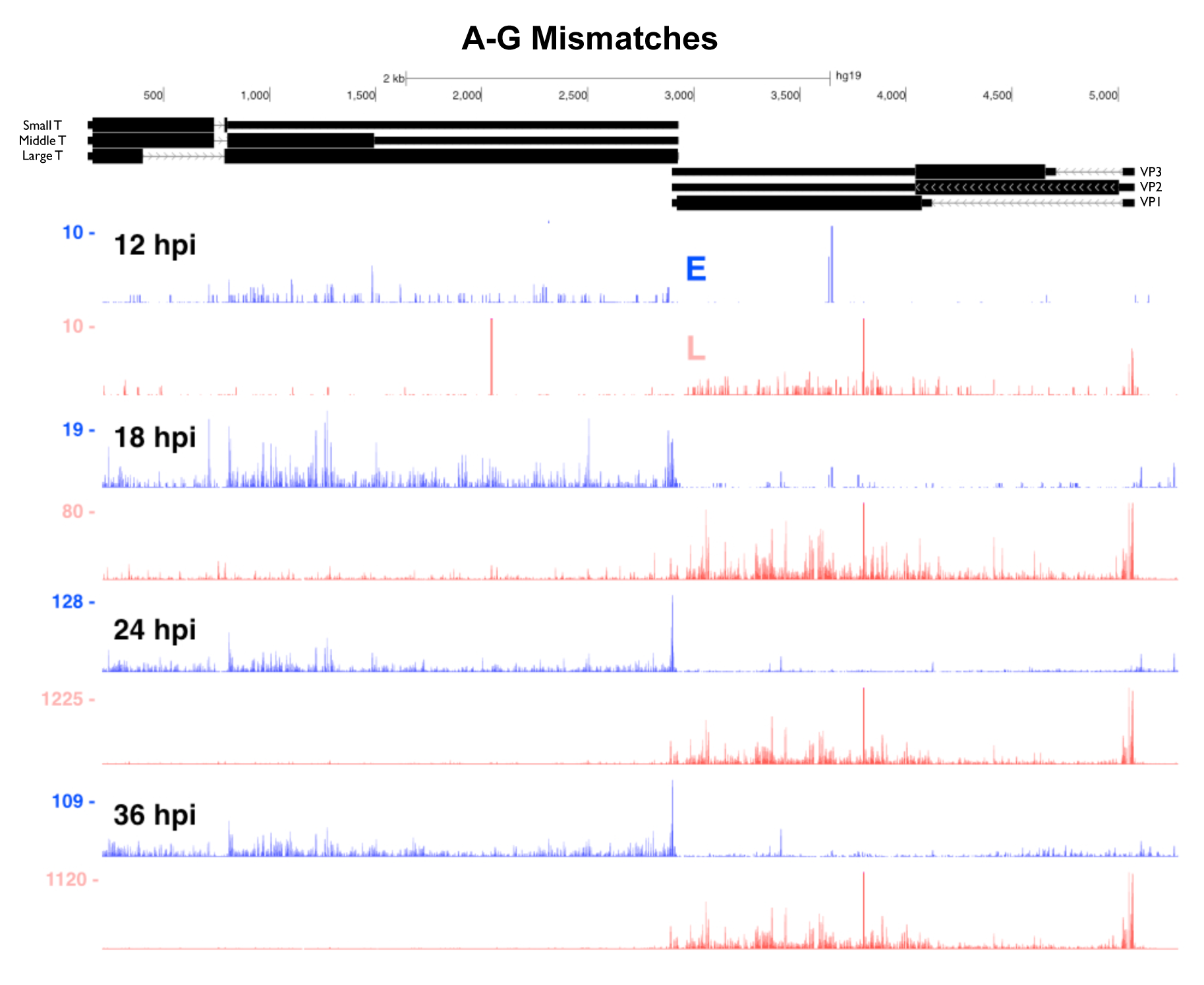

Supplement: S6 Fig — Reads from the time course run were broken from 100 bases to 25 bases and realigned with a threshold of 3 mismatches allowed per 25 base read. Reads with A-G mismatches were visualized using the UCSC genome browser to indicate regions with potential editing sites. The polyadenylation sites showed the highest number of A-G mismatched reads at late times of infection. (TIF) [file ppat.1005166.s006.tif]

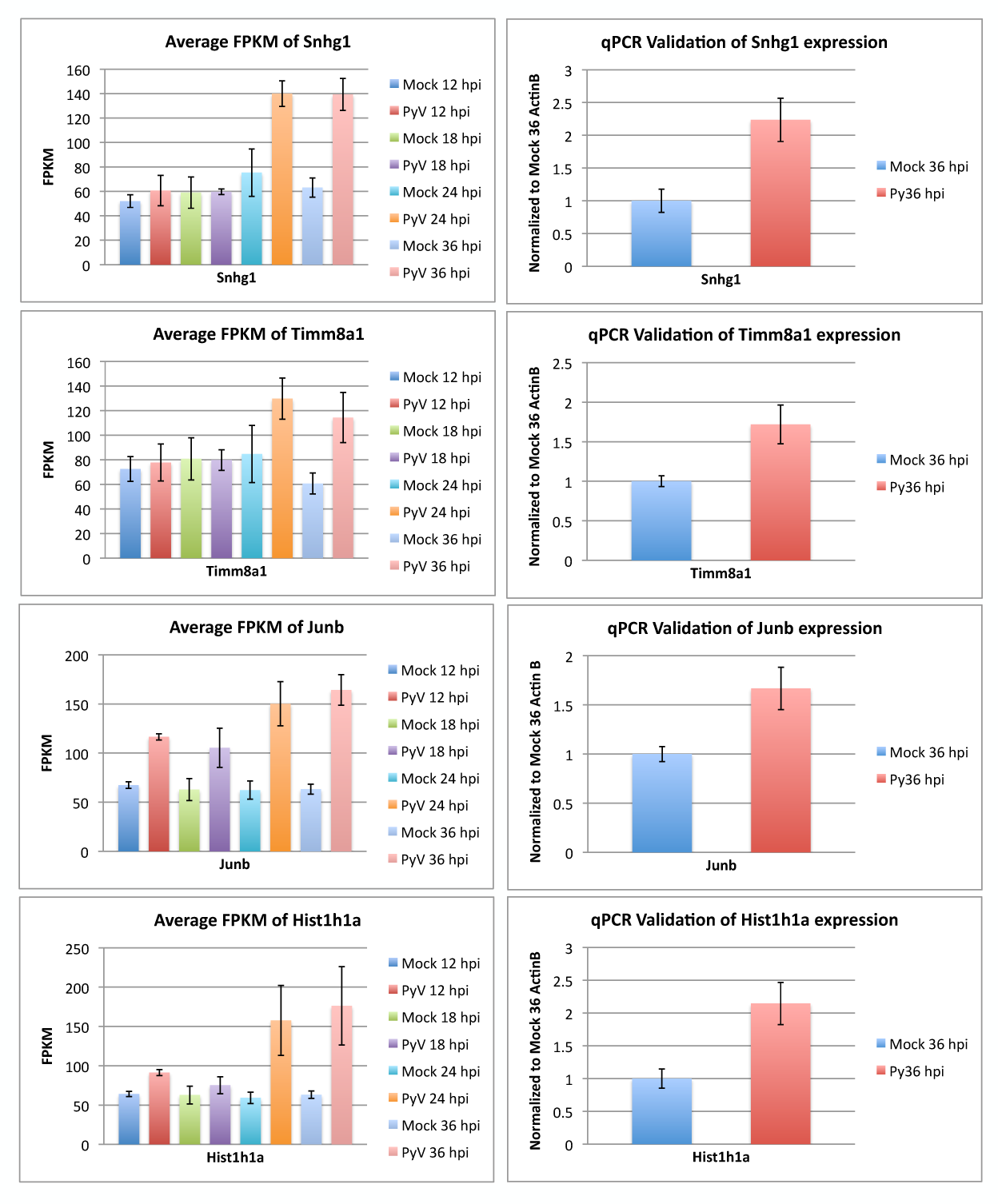

Supplement: S7 Fig — One noncoding and three coding genes with a high expression and at least 1.5 fold change were selected for validation of upregulated genes by qPCR. FPKM results shown in left column. qRT-PCR results shown in the right column. (TIF) [file ppat.1005166.s007.tif]

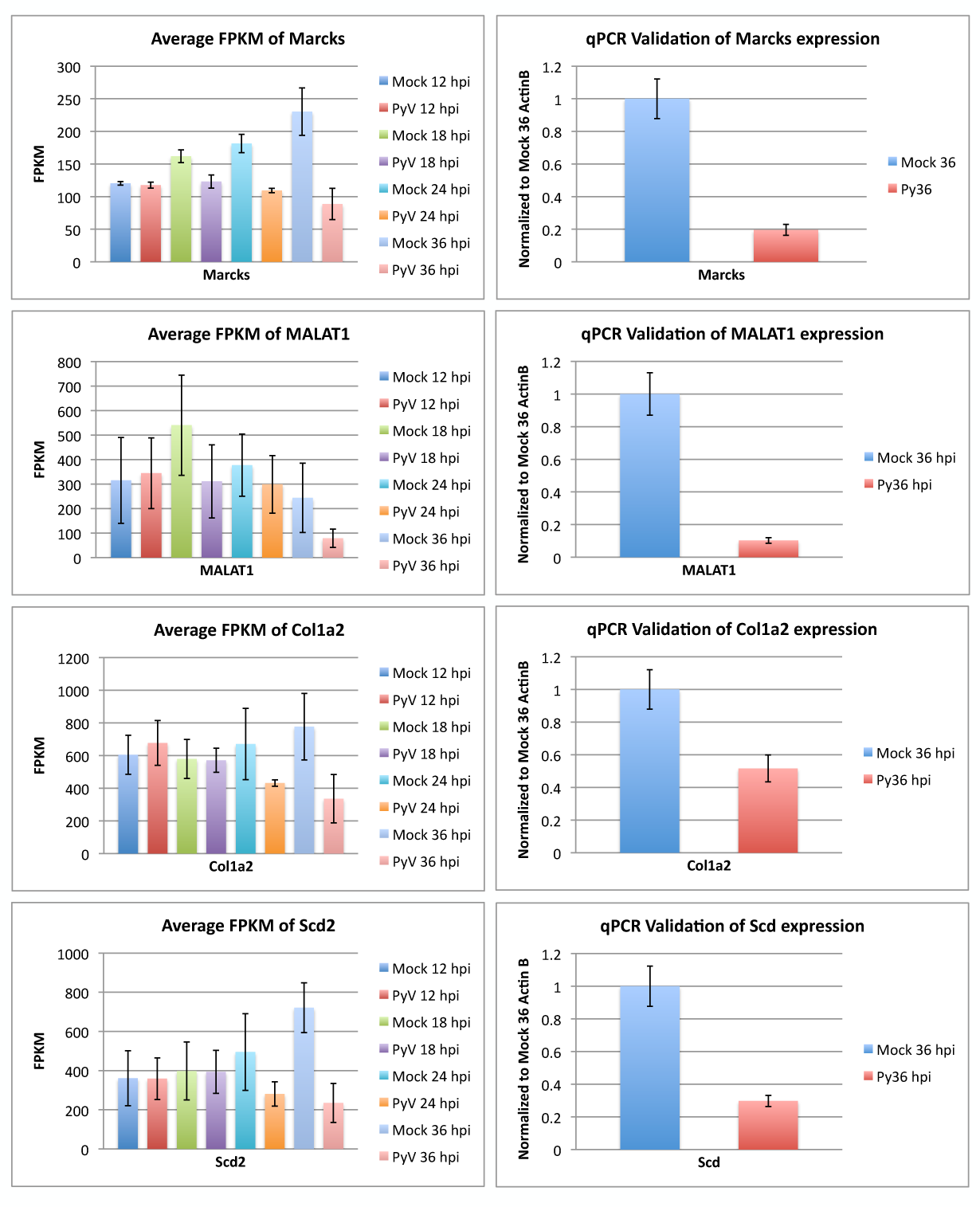

Supplement: S8 Fig — One noncoding and three coding genes with a high expression and at least 1.5 fold change were selected for validation of downregulated genes by qPCR. FPKM results shown in left column. qRT-PCR results shown in the right column. (TIF) [file ppat.1005166.s008.tif]

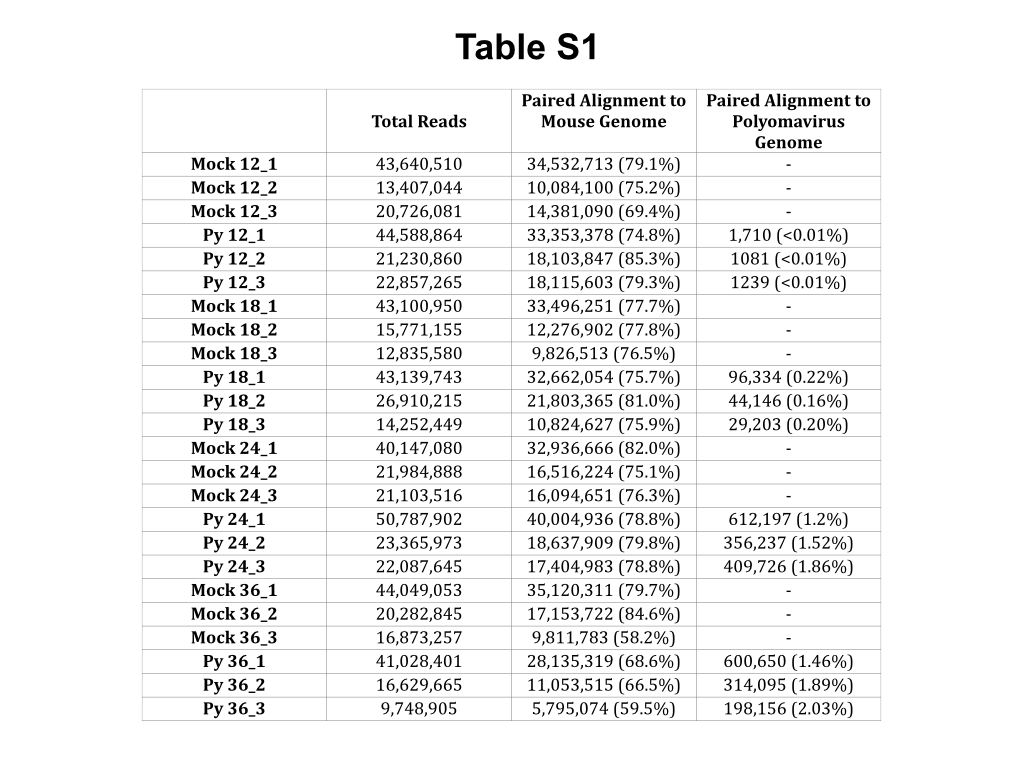

Supplement: S1 Table — Reads from the three replicates of each time point in the time course. Total reads shown in the left column. Number and percentage of reads aligned to the mouse genome shown in the middle column. Number and percentage of reads aligned to the virus genome shown in the right column. (TIFF) [file ppat.1005166.s009.tiff]

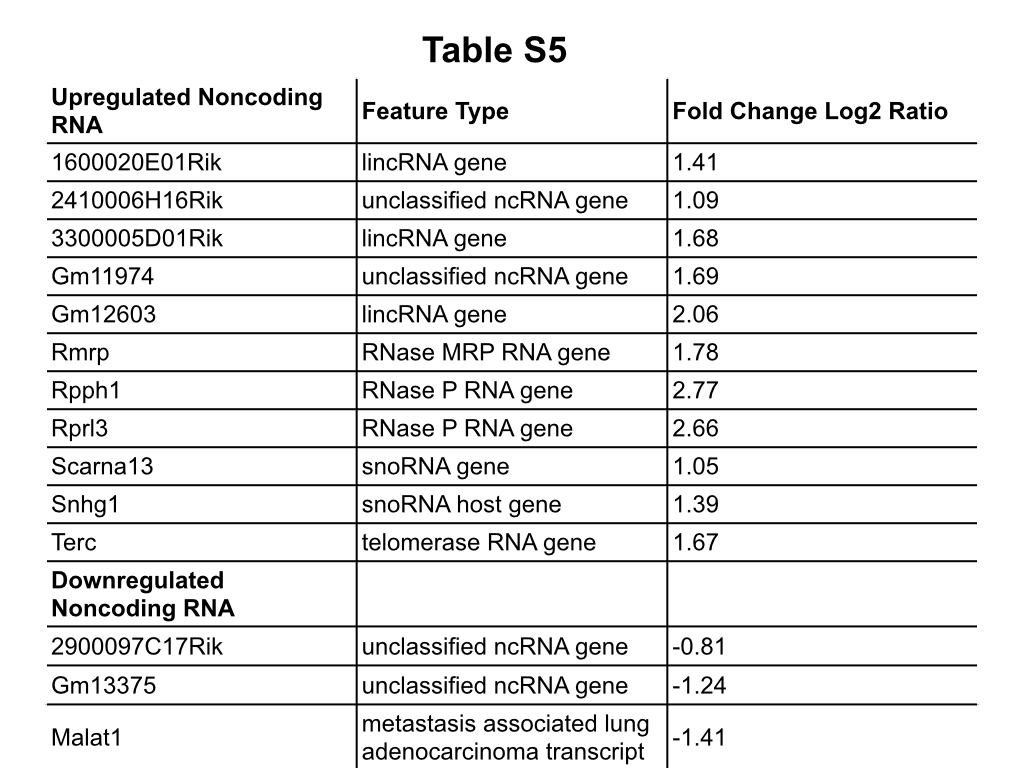

Supplement: S5 Table — List of noncoding RNAs by name, feature type, and fold change log2 ratio that differs more than 1.5 fold between Mock and PyV infected samples by 36 hours after infection. (TIFF) [file ppat.1005166.s013.tiff]
